# A national-scale assessment of the impact of canals on urban temperatures

**DOI:** 10.1038/s41467-026-75731-0

**Published:** 2026-08-10

**Authors:** M. D. Tomkins, H. McDonald, J. J. Huck, J. Tippett

**Affiliations:** 1 MCGIS, Department of Geography, School of Environment, Education and Development, The University of Manchester, Manchester, UK; 2 Department of Electrical & Electronic Engineering, School of Engineering, The University of Manchester, Manchester, UK; 3 Department of Planning, Property and Environmental Management, School of Environment, Education and Development, The University of Manchester, Manchester, UK

**Keywords:** Environmental sciences, Climate-change mitigation, Geography

## Abstract

Urban populations are particularly vulnerable to heat-related mortality and morbidity. Climate-responsive urban design, which can include development of green and bluespaces, has the potential to offset climate change-related impacts. Compared to greenspaces, the effects of bluespaces on urban temperatures have received comparatively little attention, with uncertainty regarding the magnitude of urban cooling. In this study, we develop an energy balance model to estimate the thermal effects of canals and apply this to the canal network of Great Britain and Ireland. Here we show that the modelled canals provide an average annual daytime cooling effect of ~0.8 °C (0.7 – 1 °C) within the immediate vicinity of the canal network, with more pronounced cooling during spring and early summer (~1.2 – 1.4 °C) and during heatwaves (~1.5 – 2.3 °C). While thermal effects are not exclusively positive, our results indicate that canals can improve thermal comfort and reduce heat risk, with related benefits for population health and wellbeing.

## Abstract (web summary)

Climate-responsive urban design aims to create cooler and more liveable cities. This study shows that canals can cool their immediate surroundings, particularly during heatwaves, with related benefits for population health and wellbeing.

## Introduction

Anthropogenic climate change is one of the principal threats facing humanity^1^. While climate change impacts vary in form and intensity across the globe^2^, urban areas are particularly vulnerable due to the high and growing concentration of human assets and activities^3,4^, comprising ~4.5 billion individuals in 2022^5^. Impacts associated with rising average temperatures, as well as an increase in the frequency and intensity of extreme weather events, such as heatwaves^6^, also have a disproportionate impact in urban areas due to the urban heat island (UHI) effect, in which cities are typically warmer than surrounding rural areas due to the effect of building materials and morphology on energy exchange and airflow^7,8^. In combination, these trends have important consequences for wellbeing, morbidity and mortality in urban areas^9^.

While urgent global-scale climate action is ultimately required to target the underlying drivers of climate change^1^, adaptation strategies enacted at the local scale, such as modifications to urban design, have the potential to mitigate against severe climate change impacts^10^. The presence of greenspace (such as urban forests, parks or green roofs) is one commonly studied example and has been shown to reduce urban temperatures by ~0.94 °C during the day^11^. By comparison, the effects of bluespaces (such as rivers, lakes, and canals) on urban temperatures have received comparatively little attention^12^, with uncertainty regarding the magnitude of urban cooling^13,14^. This uncertainty stems in part from differences in study design and the varying strengths and limitations of remote sensing, on-site measurement, and modelling approaches^13,15^. For example, while micrometeorological simulations^16^ might incorporate a range of environmental characteristics (e.g., building materials, vegetation) and/or hydrological–atmospheric processes, modelled at high spatial (~1–10 m) and temporal resolutions (time-steps of ~1 – 5 s), they are typically applied to a small number of sites for limited durations (~24–48 h). In contrast, while remote sensing datasets enable long-term analysis at national or even global scales, their spatial resolutions and return periods typically preclude analysis of rapidly changing processes operating over small areas.

**Fig. 1:**
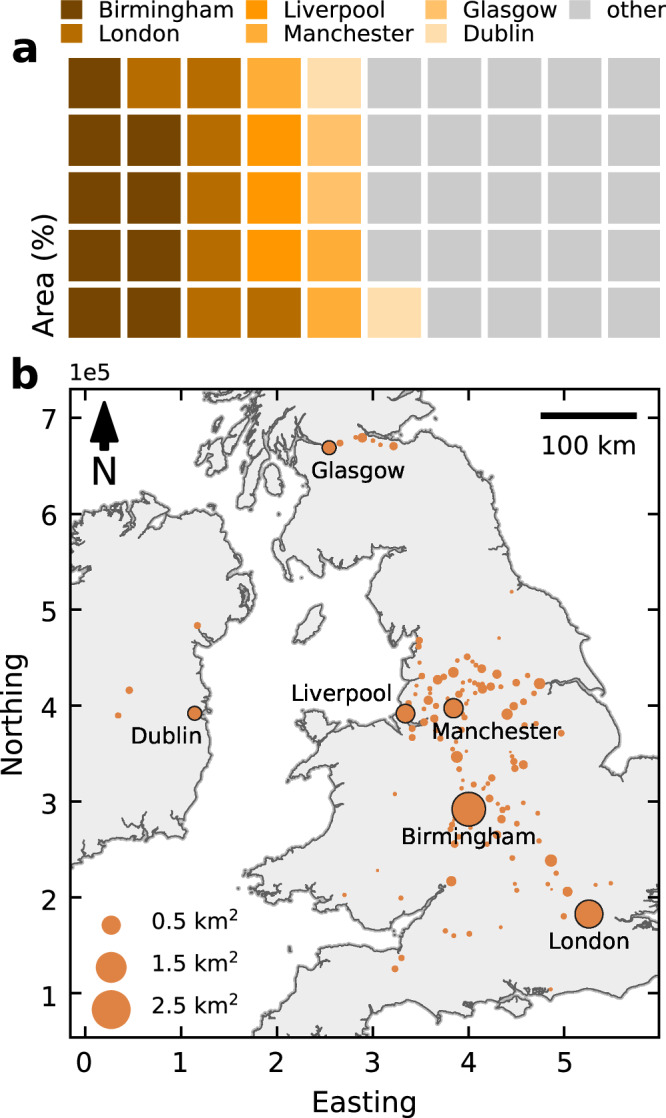
Urban canal distribution for Great Britain and Ireland. **a** Waffle chart showing the approximate canal area for the Birmingham, London, Liverpool, Manchester, Glasgow, and Dublin regions, as a proportion of the total urban canal network (one cell = 2%). **b** Spatial distribution of canal areas (km^2^) for 129 sub-regions of Great Britain and Ireland. The urban areas with the most extensive canal networks are highlighted. Collectively, these account for ~52% of the total canal area. Map projection: British National Grid.

In this study, we model the thermal effects of canals on the surrounding environment, which are a little-studied^14^ but widespread feature of post-industrial urban areas. We perform a national-scale analysis of Great Britain and Ireland, where an extensive urban canal network (Fig. 1) reflects the legacy of the Industrial Revolution^17^. Our model (see “Methods”) quantifies the energy balance of urban canals, incorporating radiative, evaporative, and conductive–convective exchanges^18^, where energy fluxes associated with water are compared to those of a reference surface (asphalt) to estimate the potential change in air temperature (Δ*PT*). The model is based upon a simplified representation of land use, incorporating the characteristics of the modelled canals (i.e., geometries, depths) and key features of the surrounding environment (i.e., weather, atmospheric shading by nearby buildings), all of which can play a key role in surface energy balance. This parsimonious approach complements computationally intensive micrometeorological simulations^16^ by enabling simulation of a complete annual cycle at a national-scale, which allows us to assess the cooling potential of urban waterways in the context of new temperature extremes^19^, and the implications of urban form for heat mitigation^20^.

## Results

**Fig. 2:**
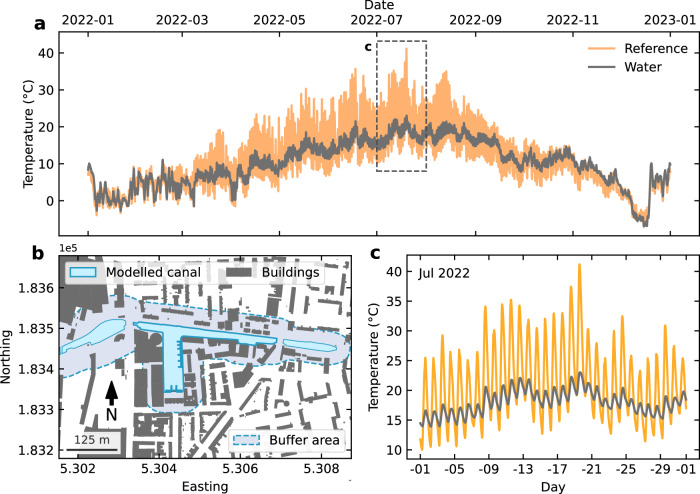
Model output for a section of the Regent’s Canal, London, UK. **a** Modelled water (*wT*) and reference temperatures (*rT*) for 2022 (15-min intervals) for a section of the Regent’s Canal, London, UK. **b** The modelled canal and nearby buildings geometries (© Crown copyright and database rights 2026 Ordnance Survey (AC0000851941)), and the buffer area used for modelling Δ*PT*. Map projection: British National Grid. **c** Modelled *wT* and *rT* for July 2022, illustrating the daily fluctuations in temperature, and the differing heat capacities of the two modelled materials.

Figure 2 shows a typical model output for one contiguous section of the urban canal network, with water (*wT*) and reference temperatures (*rT*) produced at 15-min intervals for 2022. In total, 2356 canal sections were modelled, using minimum and maximum size thresholds of 500 m^2^ and 200,000 m^2^, respectively.

### Model validation

**Fig. 3:**
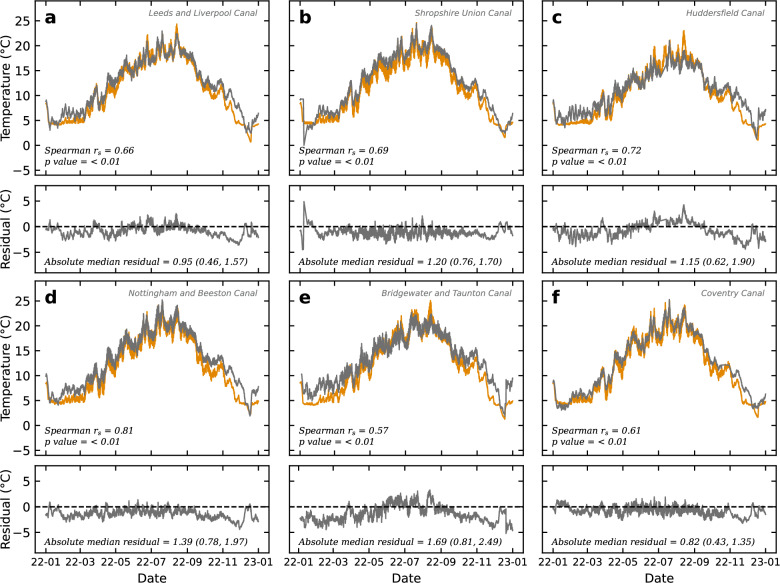
Comparison of measured vs. modelled *wT.* Comparison of modelled *wT* at 40–60 cm depth within the water column (orange lines) with measured *wT* for the same depth (grey lines), plus residuals (absolute median, *p*25, *p*75). Measured data were provided by the Canal and River Trust and were available for the Leeds and Liverpool Canal (**a**), the Shropshire Union Canal (**b**), the Huddersfield Canal (**c**), the Nottingham and Beeston Canal (**d**), the Bridgwater and Taunton Canal (**e**), and the Coventry Canal (**f**). Water temperature was measured at 10 min (**a**, **d**, **f**), 15 min (**e**), or hourly frequency (**b**, **c**). Spearman’s rank correlation coefficient (two-sided, r_s_, *p*-value) was calculated based on a stationary time series, produced using the differences between consecutive observations. Interpretation should focus on the effect size (r_s_), as large time-series sample sizes (*n* = 8516–34,727), reflecting the number of time intervals in which both measured and modelled data are present, render even weak correlations as statistically significant.

To validate our outputs, modelled *wT* was compared to measured *wT* at six locations, with data provided by the Canal and River Trust (Fig. 3). Overall, model performance is very good, with residuals commonly in the range ±1 − 2 °C and Spearman’s rank correlation coefficients of 0.57–0.81 (*p* < 0.01). Although there is evidence of some over- (e.g., Fig. 3c) and under-prediction (e.g., Fig. 3b), the models capture both seasonal variability and shorter-term fluctuations in *wT*. While additional energy fluxes could be incorporated into a future model, such as bed heat exchange, longitudinal advective heat flux or modifications during freeze-up, as well as additional site-specific information (e.g., shading by vegetation, bank materials, building albedo, wind tunnel effects), this would require a substantial increase in model complexity but would likely result in only a marginal improvement in model performance.

### Effects on air temperature

Changing water energy balance, as revealed by fluctuating water temperature (Fig. 2a), is used to predict the potential change in air temperature in the surrounding environment when the canal is present (Δ*PT*), compared to an equivalent ‘reference’ surface area of asphalt.

**Fig. 4:**
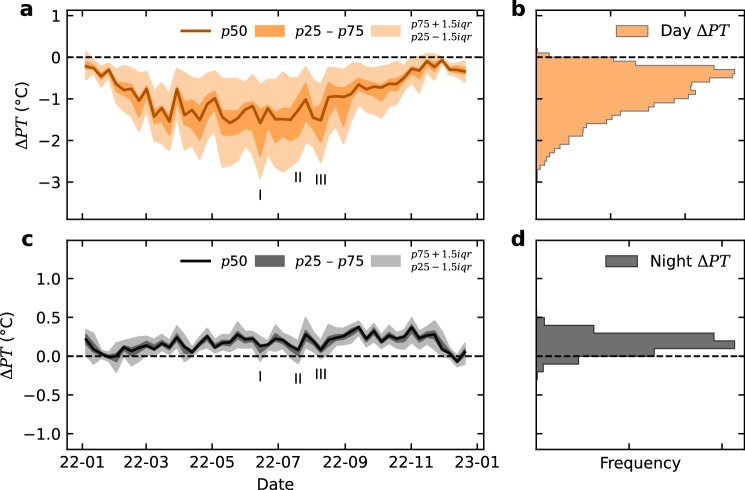
Weekly-averaged time-series of median daytime and nighttime Δ*PT.* Time-series and histograms of median weekly air temperature change (Δ*PT*) for daytime (**a**, **b**, 10:00 – 15:45) and nighttime periods (**c**, **d**, 22:00 – 03:45). Error bands in (**a**, **c**) denote *p*25 to *p*75 (dark shading) and *p*25 – 1.5 interquartile range (IQR) to *p*75 + 1.5 IQR (light shading). Designated summer heatwaves, defined as three or more consecutive days where daily maximum temperatures meet or exceed a heatwave temperature threshold^76^, occurred on 15–17 June (I), 17–19 July (II) and 9–15 August (III).

Based on this approach (see “Methods”), urban canals exert a median (*p*25, *p*75) annual daytime Δ*PT* of −0.77 °C (−1.04, −0.72) within the immediate vicinity of the canal, typically ~20–50 m from the canal banks (*p*5–*p*95), with a corresponding nighttime Δ*PT* of 0.16 °C (0.15, 0.18). This diurnal pattern of daytime cooling and nighttime warming is consistent with prior work^21,22^ and reflects the thermal inertia of water^10^. Thermal effects vary markedly across the year, however (Table 1), with the median monthly daytime Δ*PT* ranging from a minimum of −0.18 °C in November, increasing through spring (−1.19 to −1.34 °C) and early summer (−1.36 °C, Fig. 4), and reducing slightly in the later summer months (−1.05 °C to −1.20 °C). While nighttime Δ*PT* warming also follows a seasonal pattern, with maximum Δ*PT* in late summer and autumn (0.24–0.30 °C), and no clear effect in winter, in line with changing water and reference temperatures (Fig. 2a), the magnitude of nighttime warming is comparatively small.

**Table 1:** ∆*PT* (°C) statistics, summarising the median (*p*25–*p*75) daytime and nighttime ∆*PT* for annual and monthly time periods

	Median (*p*25–*p*75)	
Time period	Daytime ∆*PT* (°C)	Nighttime ∆*PT* (°C)
Annual	−0.77 (−1.04, −0.72)	0.16 (0.15, 0.18)
January	−0.35 (−0.35, −0.29)	0.06 (0.04, 0.08)
February	−0.83 (−1.14, −0.73)	0.09 (0.06, 0.11)
March	−1.19 (−1.33, −1.13)	0.15 (0.14, 0.17)
April	−1.34 (−1.70, −1.18)	0.15 (0.13, 0.18)
May	−1.29 (−1.68, −1.15)	0.19 (0.16, 0.20)
June	−1.36 (−2.05, −1.28)	0.18 (0.14, 0.21)
July	−1.20 (−1.83, −1.08)	0.19 (0.13, 0.20)
August	−1.05 (−1.54, −0.93)	0.21 (0.17, 0.21)
September	−0.73 (−0.89, −0.66)	0.30 (0.27, 0.31)
October	−0.56 (−0.70, −0.48)	0.25 (0.21, 0.26)
November	−0.18 (−0.24, −0.15)	0.24 (0.22, 0.29)
December	−0.26 (−0.31, −0.24)	0.04 (0.004, 0.05)

The magnitude of Δ*PT* also varies over much shorter timescales, reflecting the interplay of current and prior weather, as well as antecedent water temperatures. Until recently 2022 was the warmest year on record in the UK, in a series stretching back to 1884^23^ and 1659^24^, with the summer period marked by extreme heatwaves on 15–17 June, 17–19 July and 9–15 August. Of these, the 17–19 July heatwave resulted in the UK’s highest ever recorded temperature, at 40.3 °C^23^. Each heatwave is marked by an increase in median daytime Δ*PT* compared to the monthly median, reaching −1.63 °C (−2.40, −1.12), −2.33 °C (−3.03, −1.31) and −1.53 °C ( − 1.87, −1.09), respectively. While nighttime warming during the heatwaves appears negligible with respect to the reference material, there is evidence of enhanced warming in the subsequent nights, reaching 0.24 °C (0.19, 0.27), 0.24 °C (0.20, 0.27) and 0.39 °C (0.31, 0.41), respectively.

When considering temperature effects by region (Fig. 1), the magnitude of annual median daytime Δ*PT* ranges from approximately −0.32 °C to −1.37 °C within the immediate vicinity of the canal network, with variability largely explained by differences in cloudiness and windiness between regions (see Supplementary Figs. 7, 8). The largest six regions recorded daytime Δ*PT* of approximately −0.72 °C to −1.10 °C, with potential cooling benefits for ~12.4 km^2^ of the Birmingham region, ~8.1 km^2^ in London, ~4 km^2^ in both Manchester and Liverpool, and ~2 km^2^ in both Glasgow and Dublin.

### Effects on thermal comfort

To investigate the degree to which canals influence thermal comfort, we utilised a simplified version of wet-bulb globe temperature (WBGT), a thermal comfort index based on air temperature (°C) and humidity (%)^25,26^. WBGT was calculated twice, first using the input meteorological data, and second with modified air temperature data, including canal effects (+Δ*PT*). When the input meteorological data were used, and based upon existing thresholds for heat risk^25^, there was an average of ~80 h of “Moderate” heat risk in 2022 across the studied regions (≥26 °C) and ~20 h of “High” heat risk (≥28 °C). While the total number of hours of “Extreme” heat risk (≥32 °C) was relatively small, with most regions experiencing no or few hours of extreme risk (*p*95 = 3.5 h), some regions did experience elevated risk (≤49 h) in the presence of high temperature and humidity.

When air temperature data including canal effects (+Δ*PT*) were used for calculating WBGT, there is a marked reduction in the number of “Moderate” and “High” risk hours across 2022, to an average of ~33.5 h (−58%) and ~5 h (−74%), respectively. For most regions, incorporating Δ*PT* was sufficient to prevent “Extreme” heat risk entirely in the immediate vicinity of the canal (i.e., all WBGT values < 32°C). It should be recognised, however, that these estimates are based on modified air temperature alone, and do not incorporate rising humidity due to evaporation^21^, which can have an adverse effect on thermal comfort^27^. Sensitivity testing (see Supplementary Fig. 9) indicates that the canal network would still reduce the number of hours of heat risk with moderate increases in humidity (≤ 10%) but would begin to negatively impact thermal comfort beyond this.

## Discussion

Our models indicate that urban canals in Great Britain and Ireland provide a daytime median cooling effect of ~0.8°C (0.7–1 °C) across the year within the immediate vicinity of the canal network (~20–50 m from canal banks), with more pronounced cooling during spring and early summer (~1.2–1.4 °C), in line with field measurements^20^, and importantly during heatwaves (~1.5–2.3 °C). These results show that canals can contribute to urban cooling during warmer periods of the year. It should be acknowledged, however, that the effects of urban canals are not exclusively positive (i.e., Δ*PT* cooling), with evidence of nighttime warming, in line with prior work^15,21,22^, and with potential implications for nighttime heat stress^28^. The magnitude of warming is relatively small, however, in good agreement with some prior modelling^14^, with an annual median of ~0.2 °C, increasing during the late summer and autumn (~0.3 °C) and following heatwaves (~0.4 °C).

Rising urban temperatures have important consequences for population health^27^, particularly for vulnerable groups^29^, and this is likely to be exacerbated by even small changes in temperature^30,31^. In turn, partially offsetting temperature extremes, in the order of ~1–2 °C during the daytime, could have benefits for population mortality and morbidity, and could contribute to a reduction in heat-related deaths, which averaged ~800 per annum in England and Wales across 2000–2019^32^. Using the simplified WBGT thermal comfort index^25,26^, incorporating canal effects (+Δ*PT*) was sufficient to reduce the number of hours of moderate, high and extreme heat risk, under scenarios of moderate increases in humidity (≤10%). While bluespaces have been shown to increase humidity^20,33^, we anticipate only small differences within the immediate vicinity of the canal network (~20–50 m). Larger differences have been recorded elsewhere (approximately +15–20% above background rates), but only for significantly larger bluespaces, e.g., the ~1.3 km wide Yangtze River, Wuhan^34^. While further work would be required to model humidity change explicitly, or include more complex thermal comfort indexes, such as Physiological Equivalent Temperature^35^, the above approach indicates that canals have the potential to reduce heat risk.

Alongside possible physiological benefits during the daytime, there is an array of potential secondary benefits associated with the cooling effects of canals. These include the broader physical and mental health benefits that are widely acknowledged to arise from the availability, accessibility and visibility of urban bluespace^36–38^ and encouragement of active travel^39^ and access to natural spaces during warmer periods of the year, both of which have well-documented wellbeing and health benefits^40^. As rising temperatures are a key driver of uptake and use of mechanical cooling (e.g., air conditioning) in domestic and commercial settings^41,42^, particularly during heatwaves, cooling by canals could also reduce energy demand and ultimately enable carbon dioxide (CO_2_) savings. These benefits are of particular importance for Great Britain and Ireland, where ~6.2 million individuals live within 1 km of the urban canal network (~15-min walking distance; see Supplementary Discussion: Canal network accessibility).

Prior studies of the effects of urban bluespaces on temperature have utilised a range of methods (e.g., on-site measurement, modelling, remote sensing) and are typically characterised by analysis of a small number of locations, infrequent or short-term temporal sampling, and/or variability in the designation of urban reference sites^13^. This complicates comparison with the Δ*PT* values reported above, which are based on a national-scale approach, while retaining temporal breadth (annual) and resolution (15-min model intervals). It should be noted, however, that our scale of analysis is enabled by a simplified calculation of Δ*PT* based on energy transfer from the water–reference to the surrounding air in a non-spatial model and which ignores additional energy flows^15^ or spatial attributes, which may be included in analyses at smaller scales and/or durations.

While bluespace effects are comparatively little studied^14^, the model results described above are broadly consistent with prior observations, including the diurnal pattern of Δ*PT*^21^ and the seasonality of daytime Δ*PT* effects. For example, our results indicate maximum cooling during spring and early summer (Fig. 4), in line with a recent meta-analysis^43^ and in broad agreement with on-site measurements from a small urban river in Sheffield, UK^20^. Placing our modelled Δ*PT* values within the context of prior work is much more challenging, however, as differences in study design (e.g., climate zone, season of analysis, methodological approach) and bluespace characteristics (e.g., water body type, size, location) inevitably influence the magnitude of thermal impact^43^. While remote sensing approaches typically produce larger Δ*PT* estimates^13,43^, our summary values are within the range of daytime estimates^43^ produced by field measurements (mean = −1.4°C, range = +1.09 °C to −3.55 °C) and numerical modelling (mean = −1.7 °C, range = −0.1 °C to −5 °C), and indicate that even relatively narrow urban canals (Width *p*5 = 9.2 m, *p*95 = 27.2 m) can still cool their immediate surroundings.

Our models also produce Δ*PT* warming at night and during periods of higher water temperature, a phenomenon that has been showcased by prior models^21,44^ and on-site observations^20^, but which is often not addressed by remote sensing analyses^13^ and which is typically overlooked in favour of the study of daytime conditions^43^. As above, deciphering the magnitude of nighttime warming is more challenging, with some models producing more significant nighttime warming of 0.7–1.0 °C and as much as 3.5 °C depending on water temperature and other parameters^21^, while other models produce comparatively small effects^14^. Overall, while there is consensus that bluespaces can warm nighttime urban environments^10^, particularly during summer (Fig. 4c), and can degrade thermal comfort by increasing humidity^21^, the magnitude of these effects appears to be highly variable, and in general appears to be of lower magnitude than daytime cooling^14^.

In summary, there is considerable uncertainty regarding the magnitude of Δ*PT* in different environments, stemming from differences in study design and methods, including the use of remote sensing vs. on-site measurement^13,15^ and the spatial-temporal scale of analysis, as well as differences in bluespace characteristics, e.g., size and shape^43,45,46^, depth^43^, and surrounding land use^43,47^. In turn, while meta-analyses can provide insights into the likely direction of thermal impact, additional modelling or on-site observations, tailored to the characteristics of the environment, are required to assess the magnitude of Δ*PT*. Overall, our analysis indicates that urban canals can have a measurable effect on both daytime and nighttime Δ*PT*, and it is therefore essential that they are considered as part of climate-responsive urban design.

As with any numerical model, our reported *wT*, *rT* and Δ*PT* values are a reflection of the broad model design, the choice and accuracy of input data (e.g., uncertainty associated with the meteorological variables, canal depths and geometries, building geometries and heights) and the assumptions and generalisations required to represent the chosen environment and the important processes within it.

One key model choice is the selection of the reference surface or site, which has a substantial effect on the strength of the measured thermal impact^13^. Here we model the effects of canals on surrounding air temperatures in comparison with an equivalent reference area of asphalt. We regard this as a reasonable assumption for an urban environment, where high emissivity (~0.7–0.9) and low albedo (~0.1–0.2) materials are common. Moreover, given the narrow, elongate shape of the studied canals, we regard asphalt roads as the most likely alternative land-use. However, we recognise that use of reference materials with differing characteristics (e.g., albedo, emissivity, thermal conductivity, density) would influence the magnitude of Δ*PT*. For example, we would anticipate reduced Δ*PT* when the reference site includes greenspace^14^, as increased shading, evapotranspiration and albedo would influence sensible and latent fluxes and likely suppress thermal contrasts between the water and reference surface. Similarly, use of alternative materials with higher albedos (e.g., concrete) would be expected to reduce Δ*PT*. In turn, reference surface or site characteristics are likely to be a key confounding variable in any meta-analysis^13^. Future applications of the model could incorporate a range of reference materials and/or more detailed environmental characteristics to further guide urban planning. For example, modelling of vegetation cover could be used to assess net Δ*PT* e.g., the combined thermal impacts of blue– and greenspaces^48^. Similarly, modifying the reference surface to reflect varying land use in the vicinity of each modelled canal might provide further information on spatial variability in Δ*PT*.

Another important aspect is the role of scale, which has been shown to strongly influence the impact of urban green-bluespaces on human health^37^. Choice of scale, both spatial and temporal, dictates the methodological approach that can be utilised and the processes that can be represented, and perhaps most importantly, can influence our interpretation of spatial phenomena, i.e., scale-dependence^49,50^. In our model, a national-scale analysis makes it infeasible to include small-scale processes (*16*), or more detailed environment characteristics. For example, an advective flux is not included under the assumption that water flow in canals is minimal or that water temperature does not vary meaningfully within the modelled canal sections, generalisations which are supported by the correspondence between measured and modelled *wT* (Fig. 3). Similarly, while we include the effects of shading by buildings, we do not include the effects of shading by vegetation^51^ or other features of the built environment, such as the canal banks or bridges. While these (and other) aspects can be more feasibly included in small-area, short-duration models, it is unclear how representative the results of these models are at larger spatial–temporal scales. Similarly, while our model performance is good at the *chosen* spatial–temporal scale (Fig. 3), its applicability to alternative scales is unknown, such as extending the model to future years, modelling at a higher temporal resolution, or evaluating performance spatially, e.g., comparison of modelled Δ*PT* with empirical measurements at corresponding locations on the canal network. More broadly, the potential impact of scale raises questions about how estimates produced by different methods (e.g., on-site observations, numerical modelling, remote sensing), all of which can be produced at a range of scales, should be reliably combined.

One aspect of scale that is particularly important in our model is the choice of bluespace cooling distance, which is used to estimate the mass of air (*m*) surrounding the canal in which thermal effects are likely to be felt (see “Methods”). While there is a relationship between bluespace width and cooling distance, which is reflected in our analysis and quantified based on field measurements^20,52^, the magnitude of Δ*PT* is intrinsically linked to the choice of distance, with larger distances resulting in smaller Δ*PT* values and vice versa (see Supplementary Discussion: Model design, for sensitivity testing). While some studies provide estimates of the cooling distance^43^, this can vary significantly, for example, due to bluespace size and geometry^53^, building density and structure^54^, and wind speed and direction^52^. While the choice of cooling distance, and other key model attributes described above, will inevitably influence model performance, the modelled Δ*PT* values appear reasonable within the context of published literature^43^, showcase behaviour that is consistent with on-site measurements and physical principles e.g., daytime cooling, nighttime warming^10^, and provide a valuable quantitative reference for the microclimate effects of urban canals, which can be tested further via micrometeorological simulations or field-based measurements.

Within this context, this research has developed and tested a methodological approach, building an energy balance model that is sufficiently parsimonious to enable analysis at a national scale and over an entire annual cycle. This approach combines physically based equations, empirical formulae and spatial analysis to produce key insights into the role of bluespaces in cooling urban areas, building upon observations gleaned from small-area, short-duration models or empirical measurements. Our results indicate that the canals of Great Britain and Ireland exert a small but significant thermal influence on their immediate surroundings, which must be considered as part of climate-responsive design. This influence is generally positive (−Δ*PT*), with typical daytime cooling benefits of ~0.7–1 °C, increasing during spring and early summer (~1.2–1.4 °C). Canals also provide important cooling benefits during periods of extreme heat (~1.5–2.3 °C), which is of critical importance if the exceptional heatwaves of recent years^55^ become routine in future decades. As widespread features of post-industrial landscapes (Fig. 1), and accessible to millions of people, lower temperatures during periods of elevated heat risk could be sufficient to improve thermal comfort, minimise heat stress, and ultimately reduce population mortality and morbidity. Our results also show that canals, which were an essential part of the early stages of the Industrial Revolution and the societal transformations that followed, are still providing tangible benefits to urban populations today.

## Methods

### Model design

Our model is used to predict Δ*PT* when a canal is present, compared to an equivalent reference surface. This is achieved using a mixture of physically based equations and verified empirical formulae, as well as carefully considered assumptions, to quantify the relevant latent, sensible (conductive–convective) and radiative (solar–thermal) fluxes between the material (water or reference) and the outside environment.

### Energy balance

For modelling the energy balance of the water and changes in temperature (Fig. 1), we use the following approach:

1 $${Q}_{n}=\pm {Q}_{r}\pm {Q}_{e}\pm {Q}_{h}$$

where ${Q}_{n}$ = total net heat exchange, ${Q}_{r}$ = heat flux due to net radiation, ${Q}_{e}$ = latent heat flux due to evaporation and condensation, and ${Q}_{h}$ = heat flux due to sensible transfer between the air and the surface. This is a simplified version of earlier modelling of the river heat budget^56^, excluding heat flux due to bed conduction (${Q}_{{hb}}$), heat flux due to friction (${Q}_{{fc}}$), and heat flux due to advective transfer in precipitation and groundwater (${Q}_{a}$). While these fluxes can be important in certain settings^56^, the correspondence between measured and modelled water temperatures (Fig. 3) indicates that the heat budget of the studied canals can be adequately accounted for by latent, sensible, and radiative fluxes alone. Exclusion of ${Q}_{{fc}}$ and ${Q}_{a}$ is justified based on the assumption of minimal water flow within the canal network, while data were not available to rigorously estimate ${Q}_{{hb}}$. The energy exchange between the water surface and surrounding air (*Q*_*n*_) is dependent upon the surface water temperature, which is influenced by the temperature distribution within the water column. To account for this, the water is modelled as a stratified system, with the canal divided into vertical layers, with corresponding conductive–convective exchanges. This design reflects the stratification that is likely in water bodies with minimal water flow, but the underlying assumptions would be invalid for water bodies with higher flow velocities, and where additional energy fluxes would be required (e.g., ${Q}_{{fc}}$).

For modelling the heat budget of the reference surface, a similar approach to Eq. (1) is used:

2 $${Q}_{n}=\pm {Q}_{r}\pm {Q}_{h}$$

excluding the latent heat flux (${Q}_{e}$). While ${Q}_{e}$ could be important in certain instances, e.g., evaporation of water from the reference surface following precipitation, it is not feasible to model at our scale of analysis.

### Net radiation

For modelling ${Q}_{r}$, incoming solar radiation was estimated using PySolar^57^, incorporating the effects of cloud cover^58^:

3 $$I=1-0.00243C-\left(4.24\cdot {10}^{-5}\right){C}^{2}$$

where *I* = fraction of clear sky irradiance expected and *C* = percentage of cloud cover in the sky. Non-solar radiative thermal absorption (*a*) was modelled incorporating cloud height (*K*), cloud cover (*C*), air temperature (*T*_*a*_) and relative humidity (*RH*), following^59^, using a fixed value of *K* = 0.06 to represent cloud heights > 5 km:

4 $$a=\left[1+{{KC}}^{2}\right]\cdot 8.78\cdot {10}^{-13}\cdot {{T}_{a}}^{5.852}\cdot {{RH}}^{0.07195}$$

Thermal emissions (*e*) were estimated following the Stefan–Boltzmann law, where *ε* = surface emissivity, *σ* = Stefan–Boltzmann constant and *T*_*s*_ = surface temperature.

5 $$e=\varepsilon \sigma {{T}_{s}}^{4}$$

### Latent flux

For modelling ${Q}_{e}$, the evaporation–condensation rate was calculated using a Penman-type empirical equation following^56^:

6 $${E}_{v}=0.165\cdot \left(\right.0.8+(0.864\cdot U)\cdot \left({E}_{w}-{E}_{a}\right)$$

where *E*_*v*_ = evaporation/condensation rate, *U* = wind speed, *E*_*w*_ = saturated vapour pressure at water surface temperature and *E*_*a*_ = vapour pressure at air temperature.

### Sensible flux

For modelling *Q*_*h*_ for water, a formulation developed by^60^ was used:

7 $${Q}_{h}={p}_{a}{c}_{{pa}}\left(c+d\cdot U\right)\left({T}_{a}-{T}_{s}\right)$$

where *p*_*a*_ = air density and *c*_*pa*_ = the specific heat of air. The empirical constants *c* and *d* are defined as $c=1.99\cdot {10}^{-3}$ (m s^−1^) and $d=2.13\cdot {10}^{-3}$, following^60^.

For modelling the equivalent flux ${Q}_{h}$ for the reference surface (via forced convection), heat transfer was calculated using an empirical correlation for laminar flow over a flat plate as follows:

8 $${Q}_{h}=h\cdot \left({T}_{a}-{T}_{s}\right)$$

where *h* = heat transfer coefficient, calculated as follows:

9 $$h=0.664\cdot {T}_{c}\cdot {\Pr }^{\frac{1}{3}}\cdot {U}^{\frac{1}{2}}\cdot {v}^{-\frac{1}{2}}$$

where *T*_*c*_ = thermal conductivity, *Pr* = Prandtl number and *v* = kinematic viscosity.

### Calculating Δ*PT*

For calculating Δ*PT*, we use the following equation:

10 $$\triangle {PT}=\frac{{Q}_{{hw}}-{Q}_{{hr}}}{{mc}}$$

where the numerator reflects the net sensible heat exchange with the air i.e., the difference between the sensible flux associated with water (${Q}_{{hw}}$) and the equivalent flux associated with the reference surface (${Q}_{{hr}}$), *m* is the mass of the surrounding air, and *c* is the specific heat of the material (J⋅kg^−1^⋅K^−1^), with values aggregated by region (Fig. 1). It should be noted that prediction of Δ*PT* is based solely on the modelled energy exchange from the water or reference to the surrounding air in a non-spatial model, and does not take into account energy flows within the urban boundary and canopy layers^15^, as well as the degree to which these flows are modified by weather, such as changing wind^61^, or urban structure, such as canyon effects^62^. These factors could act to enhance, diminish, or influence the spatial distribution of any Δ*PT* effects.

This parsimonious approach focuses on sensible heat exchange alone and does not include ${Q}_{r}$ or ${Q}_{e}$ for estimating the direct change in air temperature. While ${Q}_{r}$ is an important component of ${Q}_{n}$, this flux does not affect the temperature of the air directly and only does so indirectly by (1) modifying the temperature of the surface, or (2) through absorption by gases in the air. While ~90% of the total longwave radiation emitted by the Earth’s surface is ultimately absorbed by the atmosphere^63^, only a very small proportion is likely to be absorbed in the narrow band of air immediately above the surface, which is the focus of our model. For example, we would expect longwave infra-red absorption of just 0.0035% in a span of 10 metres, using an equivalent band average coefficient of 0.35 km^−1^ ^64^. While ${Q}_{r}$ could be included in future calculations of Δ*PT*, this would require a number of assumptions, such as the composition of the air or information on the spectral distribution of emitted radiation, or computationally intensive line-by-line radiative transfer modelling.

Similarly, the latent flux (${Q}_{e}$) is a critical component of ${Q}_{n}$^56^, without which modelled water temperatures deviate significantly from the measured values (see Supplementary Fig. 1). However, ${Q}_{e}$ is the energy flux associated with phase transitions (i.e., evaporation or condensation), rather than a direct change in temperature. In our model, ${Q}_{e}$ indirectly influences Δ*PT* through evaporative cooling of the water body and modification of the surface-air temperature gradient, influencing ${Q}_{h}$^14^.

In Eq. (10), the coefficient *m* represents the mass of the surrounding air, and in combination with the specific heat capacity of the air (*c*), directly determines the magnitude of Δ*PT*. To estimate *m*¸ we use a 10-m tall buffer around each canal section (e.g., Fig. 2b), where the buffer area is a function of the relationship between bluespace width and empirically-derived cooling distances^53,54^, using field measurements from Sheffield, UK^20^ and Hiroshima, Japan^52^. This approach (see Supplementary Discussion: Model design, for further detail and sensitivity testing) produces a median cooling distance of 27.1 m (*p*5 = 20.6 m, *p*95 = 47.9 m, range = 11.5–260 m), which is consistent with prior studies that have shown temperature effects within ~30–50 m of small water bodies^20,54,65,66^, and greater cooling distances for wider rivers^52^ and lakes^67,68^.

### Model implementation

An initial model spin-up was performed for each canal section to generate starting *wT*, *rT* and corresponding energy values (2022–01–01 00:00:00; see Supplementary Fig. 2). This produced a starting surface *wT* of ~8.9°C, which is consistent with empirical measurements by the Canal and River Trust for the same datetime (9.0 ± 0.5°C, Mean ± SD; Supplementary Table 1).

Once complete, the model was iterated at 15-min intervals for 2022 to produce new *wT* and energy values for each layer of the water column, which is modelled based on a given canal depth, subdivided into 0.2 m layers. Water–reference energy balance was modelled using sensible^60^, latent^56^, and radiative^58–60,69^ exchanges, as outlined in Eqs. 1–10, utilising the weather data described above and the characteristics of the environment, including atmospheric shading by nearby buildings.

### Analysis

When each section had been modelled, energy fluxes were aggregated by region (Fig. 1), and Δ*PT* was calculated based on the sensible energy exchange from the total canal area (${Q}_{{hw}}$) to the surrounding air (*m*), compared to the equivalent exchange from a ‘reference’ surface area of asphalt (${Q}_{{hr}}$). To assess changing Δ*PT* through time, we extracted values corresponding to the core periods of daytime (10:00 – 15:45) and nighttime (22:00–03:45) to provide a consistent basis for comparison across the year and returned summary statistics for a range of time periods.

### Reporting summary

Further information on research design is available in the Nature Portfolio Reporting Summary linked to this article.

## Supplementary information

Supplementary Information

Reporting Summary

Transparent Peer Review file

## Data Availability

The model utilises hourly weather data provided by OpenWeather^70^, including air temperature (K), humidity (%), cloud cover (%), wind speed (m/s) and air pressure (hPa), interpolated at 15-min intervals for model stability. For Great Britain, canal characteristics (geometries, depths) were provided by the Ordnance Survey (OS) and the Canal and River Trust. Canal geometries were filtered to urban areas based on the UK Centre for Ecology & Hydrology (UKCEH) 2021 Land Cover Map^71^ and were pre-processed to produce contiguous canal sections (e.g., Fig. 2b). Building characteristics (geometries, heights) were obtained from the OS MasterMap Topography layer^72^. For Ireland, canal characteristics and building geometries were obtained from OpenStreetMap^73^. Canal geometries were filtered to urban areas based on either the UKCEH 2021 Land Cover Map for Northern Ireland^71^ or the CORINE 2018 Land Cover Map for the Republic of Ireland^74^. Building heights for Ireland were derived from modelling by Keany et al.^75^. As many of these datasets are proprietary, it is not possible to provide them in full. While example datasets have been provided in the code repository to allow for model testing (see Code Availability), users would need to utilise open-source alternatives to apply the models more widely, e.g., OpenStreetMap^73^.

## Code availability

To facilitate wider use and testing of our model, the full implementation is made available on Git: https://github.com/matt-tomkins/national-canal-cooling. This methodology could be readily applied to similar bluespaces globally or could be adapted to those with differing characteristics, e.g., inclusion of ${Q}_{{fc}}$ and ${Q}_{a}$ for analysis of urban rivers.
