# Supplementary material for: A national-scale assessment of the impact of canals on urban temperatures
Source: Nat Commun. 2026 Aug 10;17:7849. doi: 10.1038/s41467-026-75731-0 (PMC13458440; doi:10.1038/s41467-026-75731-0)
Supplement: Supplementary file 1 [file 41467_2026_75731_MOESM1_ESM.pdf]

SUPPLEMENTARY INFORMATION

A national-scale assessment of the impact of canals on urban temperatures

Tomkins, M.D*[1]., McDonald, H[2]., Huck, J.J[1]., Tippett, J[3].

[1]MCGIS, Department of Geography, School of Environment, Education and Development, The University of Manchester, [2]Department of Electrical & Electronic Engineering, School of Engineering, The University of Manchester, [3]Department of Planning, Property and Environmental Management, School of Environment, Education and Development, The University of Manchester

*corresponding author, matthew.tomkins@manchester.ac.uk

List of Supplementary Items

Supplementary Note: Additional Figures and Tables

Supplementary Figure 1 The impact of latent heat (Q_e) on modelled water temperatures

Supplementary Figure 2 Results of a 30-day model spin-up for a section of the Regent's Canal, London, UK.

Supplementary Table 1 Starting water temperatures (wT) at 01/01/2022 (00:00:00).

Supplementary Discussion: Model design

Supplementary Figure 3 Map of the canal network and modelled buffer area, Birmingham

Supplementary Figure 4 Comparison of bluespace geometries and cooling distances (m)

Supplementary Figure 5 Sensitivity testing of the effects of bluespace cooling distance on ΔPT

Supplementary Discussion: Air temperature effects by region

Supplementary Figure 6 Histogram of annual daytime ΔPT values for each region

Supplementary Figure 7 Scatter plot of region attributes vs. median daytime ΔPT

Supplementary Figure 8 Scatter plot of region attributes vs. median nighttime ΔPT

Supplementary Discussion: Sensitivity testing of wet-bulb globe temperature

Supplementary Figure 9 Effects of ΔPT and changing humidity on modelled wet-bulb globe temperature

Supplementary Discussion: Canal network accessibility

Supplementary Table 2 Population within a 15-minute walking distance of the canal network (~1 km), subset by region.

Supplementary Discussion: Short-term model performance

Supplementary Figure 10 Comparison of measured vs. modelled *wT* for 1st March 2022

Supplementary Figure 11 Comparison of measured vs. modelled *wT* for 1st June 2022

Supplementary Figure 12 Comparison of measured vs. modelled *wT* for 1st September 2022

Supplementary Figure 13 Hourly absolute model residuals for measured vs. modelled *wT*

Supplementary References

Supplementary Note: Additional Figures and Tables

The following section includes additional figures to support key points in the main text.

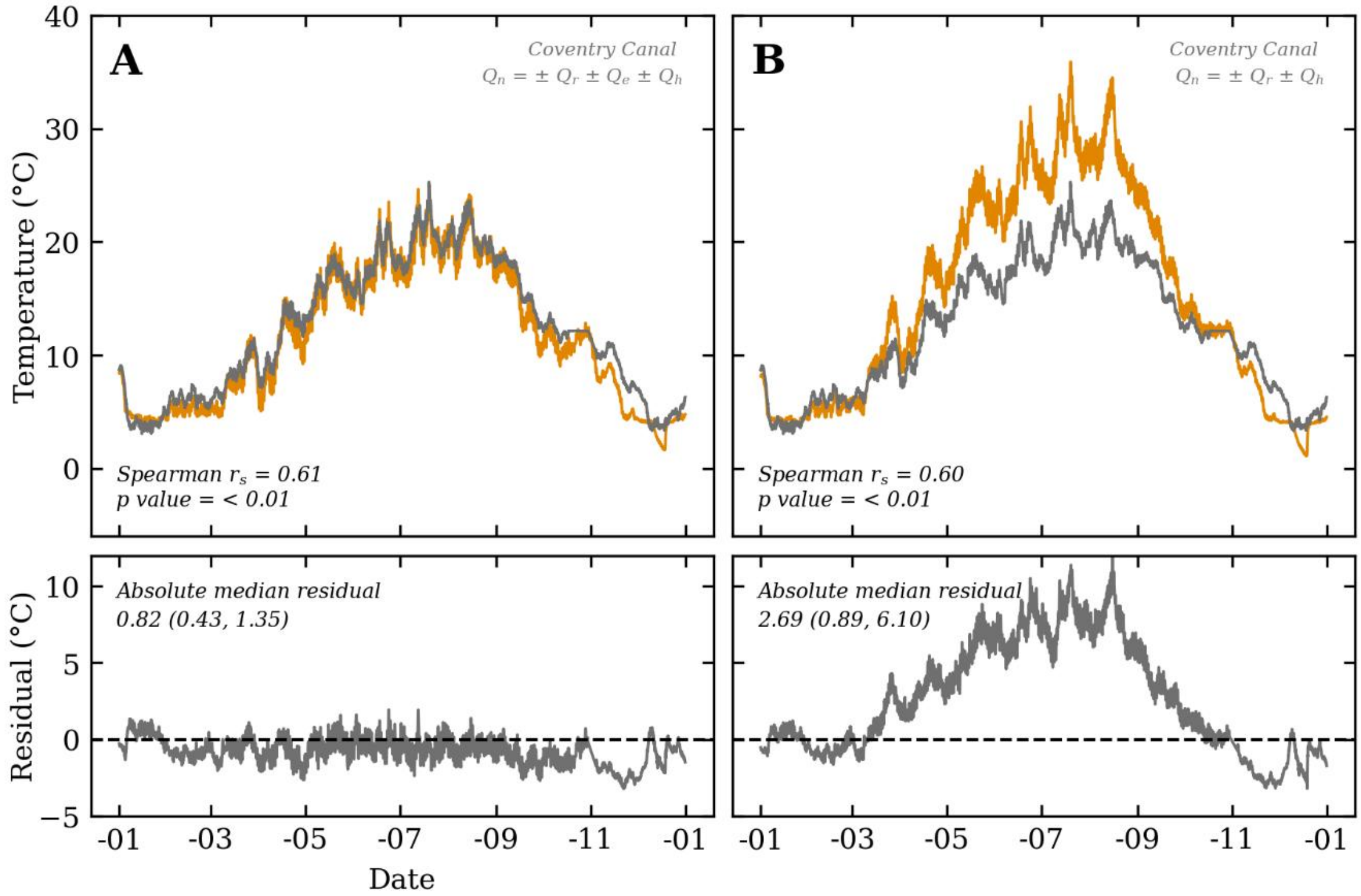

Supplementary Figure 1 The impact of latent heat (Q_e) on modelled water temperatures

Comparison of modelled wT at 40 – 60 cm depth within the water column (orange lines) with measured wT for the same depth (grey lines) for a section of the Coventry Canal, plus residuals (absolute median, *p*25, *p*75), for (**A**) the full energy balance model ($Q_n = \pm Q_r \pm Q_e \pm Q_h$) and (**B**) when the latent flux is excluded ($Q_n = \pm Q_r \pm Q_h$). Note that the Spearman's rank correlation coefficient (r_s, *p* value) was calculated based on stationary time series, produced using the differences between consecutive observations, which accounts for the similarity of the reported r_s values.

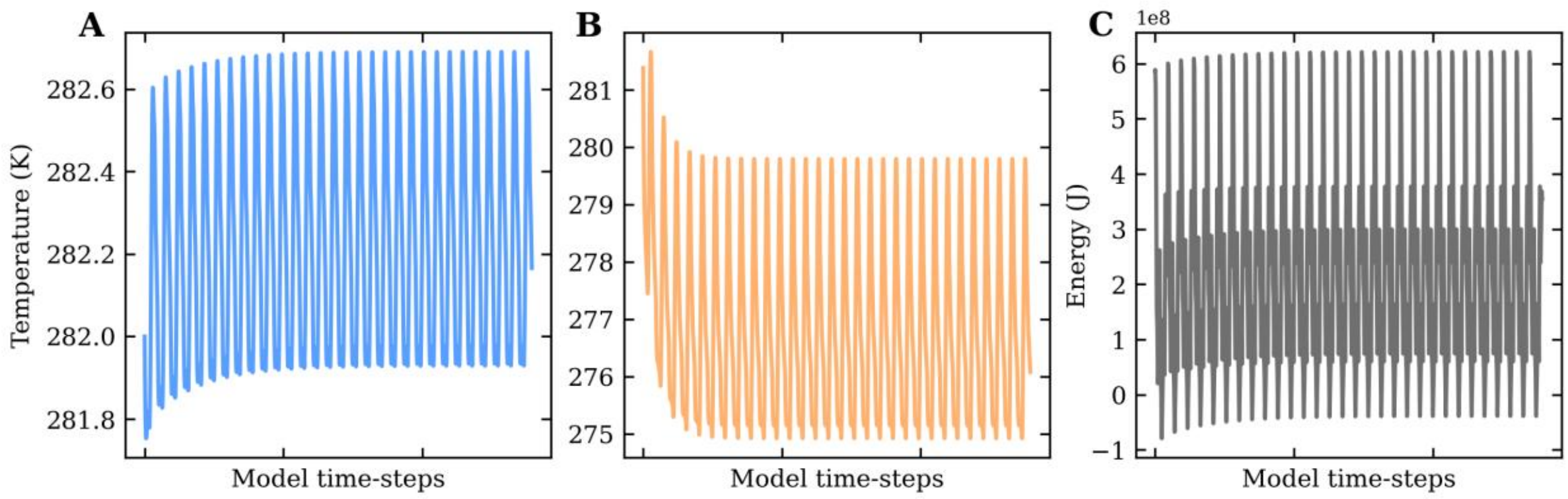

Supplementary Figure 2 Results of a 30-day model spin-up for a section of the Regent's Canal, London, UK.

(A) Modelled surface water temperatures (*wT*; K), (**B**) surface reference temperatures (*cT*; K) and **(C)** surface water energy values (*wJ*; J) for a section of the Regent's Canal, London, UK. Model spin-up utilises weather data from 15-12-2021, approximately two weeks prior to the model start date (01-01-2022). This is iterated for 30-days at 15-minute intervals (2,790 model steps) to ensure equilibrium is reached.

Supplementary Table 1 Starting water temperatures (*wT*) at 01/01/2022 (00:00:00). Data provided by the Canal and River Trust.

Sensor Name	Latitude (°)	Longitude (°)	Canal	Temperature (°C)
Bridge 73, Anchor Bridge	52.62876	-1.67918	Coventry	8.69
Bridge C, Lightbody Street	53.42274	-2.98977	Leeds and Liverpool	8.72
Bridge 4, Wakefield Road Bridge	53.64497	-1.7752	Huddersfield	8.64
Hawksbury Safety Gate	52.45793	-1.46889	Coventry	9.45
Bridge 46, Benthouse Bridge	53.64996	-2.08485	Rochdale	8.2
New Road Bridge 148	53.28674	-2.89166	Shropshire Union	9.16
Bridge 15, Chain Lane Bridge	52.93681	-1.17824	Nottingham and Beeston	9.88
Bridge, Pagefield Pipe Crossing	53.54844	-2.64974	Leeds and Liverpool	8.82
Anderton Waste Weir Flow	53.27382	-2.52924	Trent and Mersey	9.42

Supplementary Discussion: Model design

Calculating m

For calculating ΔPT, we use the following equation, as outlined in the main text:

$$\Delta PT = \frac{Q_{hw} - Q_{hr}}{mc}$$

and where m is the mass of the surrounding air. To estimate m, we use a 10-m tall buffer around each canal section, where the buffer area is a function of the relationship between the canal width and the bluespace cooling distance (*10*, *11*), an example of which is shown in Supplementary Figure 3.

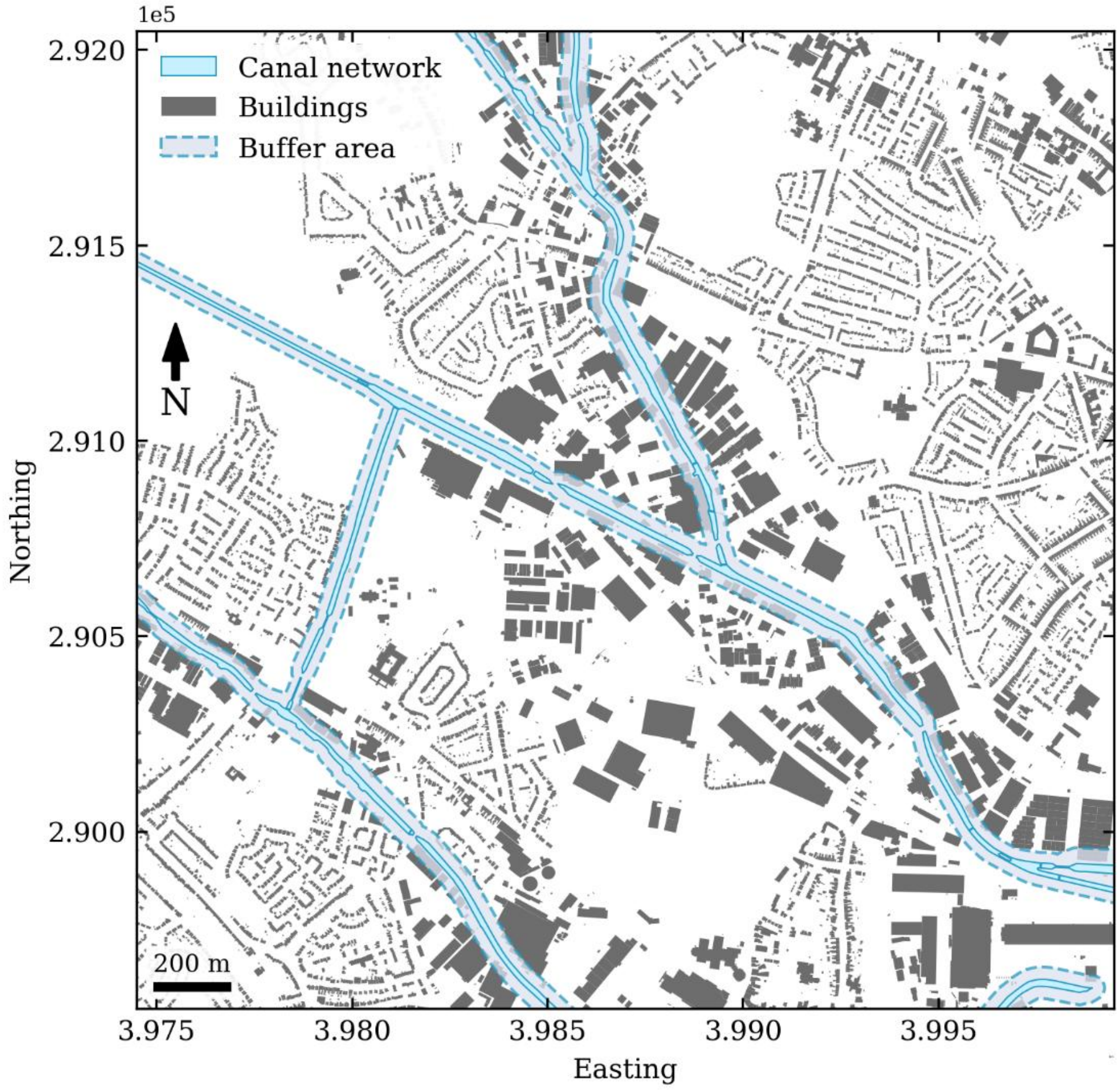

Supplementary Figure 3 Map of the canal network and modelled buffer area, Birmingham

Map of a section of the New Main Line canal, Birmingham, including the canal geometries and nearby buildings (© Crown copyright and database rights 2026 Ordnance Survey (AC0000851941)), and the modelled buffer area used for estimating ΔPT. Each contiguous canal section was buffered based on its width, based on an empirical relationship (*7*, *8*), with buffers dissolved to estimate regional ΔPT. Map projection: British National Grid

To select an appropriate buffer width for each section, we collated measurements of the bluespace cooling distance (*10*, *12–17*). In general, larger water bodies are associated with larger cooling distances (*17*), as represented by the relationship between the cooling distance and the area or root area of a bluespace, as shown in Supplementary Figure 4B. While this reveals a general correlation ($y = 1.8428 \cdot x^{0.8063}$), this approach is most appropriate for lakes and similar water bodies, which have a clearly defined area. In contrast, width is a more appropriate metric for studying elongate waterbodies like rivers and canals (*11*). Moreover, these data are primarily derived from remote sensing analyses, which typically focus on large waterbodies that are readily detectible in satellite imagery, which are not representative of the comparatively narrow canals of Great Britain and Ireland.

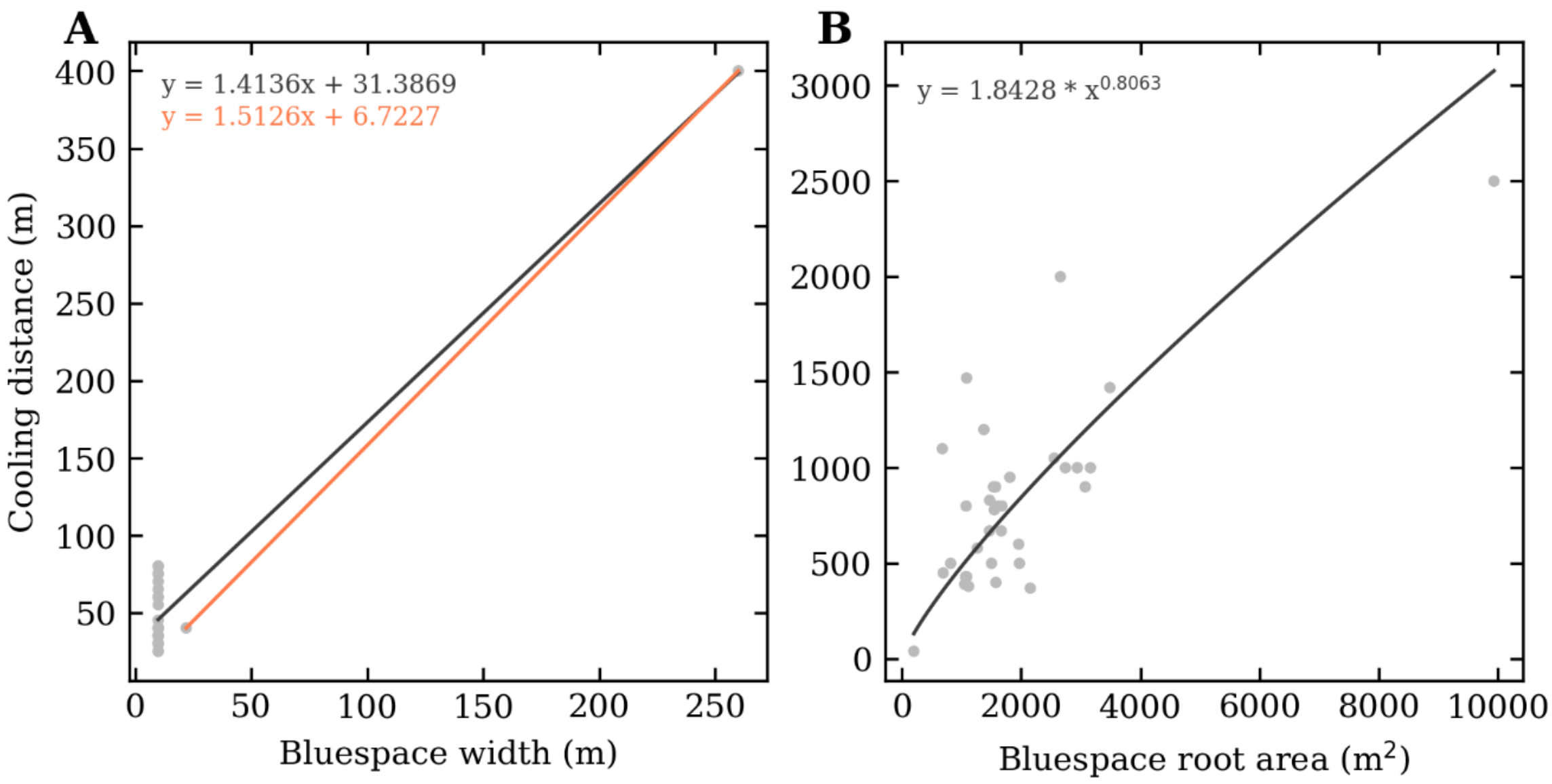

Supplementary Figure 4 Comparison of bluespace geometries and cooling distances (m)

(**A**) Scatter plot of bluespace widths (m) and cooling distances from on-site measurement in Sheffield, UK (*12*), Hiroshima, Japan (*13*) and Seoul, South Korea (*14*). Ordinary least squares regressions were produced using all the sources above (grey line, y = 1.4136x + 31.3869) and from Sheffield and Hiroshima alone (orange line, y = 1.5126x + 6.7227). (**B**) Scatter plot of bluespace root areas (m^2) and cooling distances, primarily derived from remote sensing.

Based on this rationale, we focused on cooling distances measured using on-site measurement, rather than remote sensing, and on studies of rivers or urban waterways, rather than larger lakes and wetlands (*10*, *15*). These criteria returned only a small number of relevant studies, with data from Sheffield, UK (*12*), Hiroshima, Japan (*13*) and Seoul, South Korea (*14*). An ordinary least squares regression based on this dataset is a fair approximation for the cooling distance (y = 1.4136x + 31.3869, Supplementary Figure 4A) but produces unrealistic values for narrow waterbodies due to the intercept term. By comparison, utilisation of data from Sheffield (*12*) and Hiroshima (*13*) alone produces a more realistic relationship (y = 1.5126x + 6.7227),

with a median cooling distance of 27.1 m ($p5$ = 20.6 m, $p95$ = 47.9 m, range = 11.5 – 260 m), which is consistent with prior studies which have shown temperature effects within ~30 – 50 m of small water bodies (*12*, *14*, *16*, *18*), and greater cooling distances for wider rivers (*13*) and lakes (*10*, *15*). While we regard the above empirical relationship as a reasonable estimate of the cooling distance, it is recognised that this is at best an approximation, which might vary spatially due to building density and structure (*14*), or temporally due to changing wind speed and direction (*13*).

To investigate the sensitivity of our results to changing buffer distance, we re-ran our calculation of annual ΔPT for varying distances by modifying the empirically-derived cooling distance across a range of factors [0.5 – 2.0] (Supplementary Figure 5). This modification does not impact the direction of daytime-nighttime ΔPT, but does influence the magnitude, resulting in greater daytime-nighttime effects for smaller distances, and vice versa for larger distances. While we regard the empirically-derived cooling distance as the most appropriate value, even doubling the distance (2×) would not fundamentally change our main interpretations, with a median annual daytime ΔPT of −0.42°C ($p25$ = −0.57°C, $p75$ = −0.39°C), and more pronounced cooling in spring–early summer and during heatwaves.

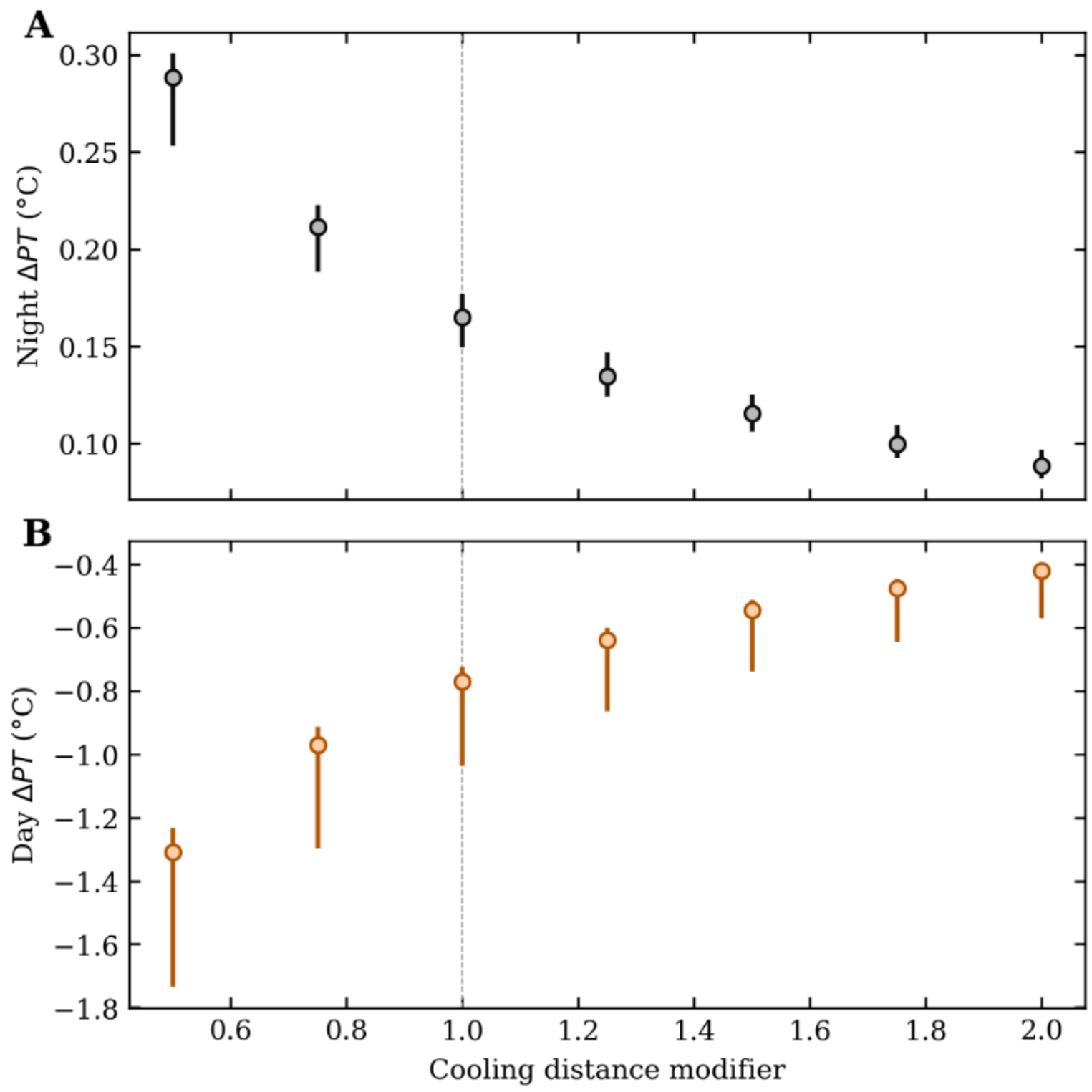

Supplementary Figure 5 Sensitivity testing of the effects of bluespace cooling distance on ΔPT

Scatter plots of annual median daytime ΔPT **(A)** and nighttime ΔPT (**B**) as a function of the cooling distance modifier, where error bars represent $p25$ to $p75$. The cooling distance was modified with respect to the default value (1×) from 0.5× to 2×.

Supplementary Discussion: Air temperature effects by region

When temperature effects are aggregated by region, the magnitude of annual median daytime ΔPT cooling ranges from ~0.32°C to ~1.37°C, as shown in Supplementary Figure 6. Potential drivers of this variability include canal geometry (e.g., width, depth), the characteristics of the surrounding environment (e.g., degree of building shading) and the weather data used to run the model.

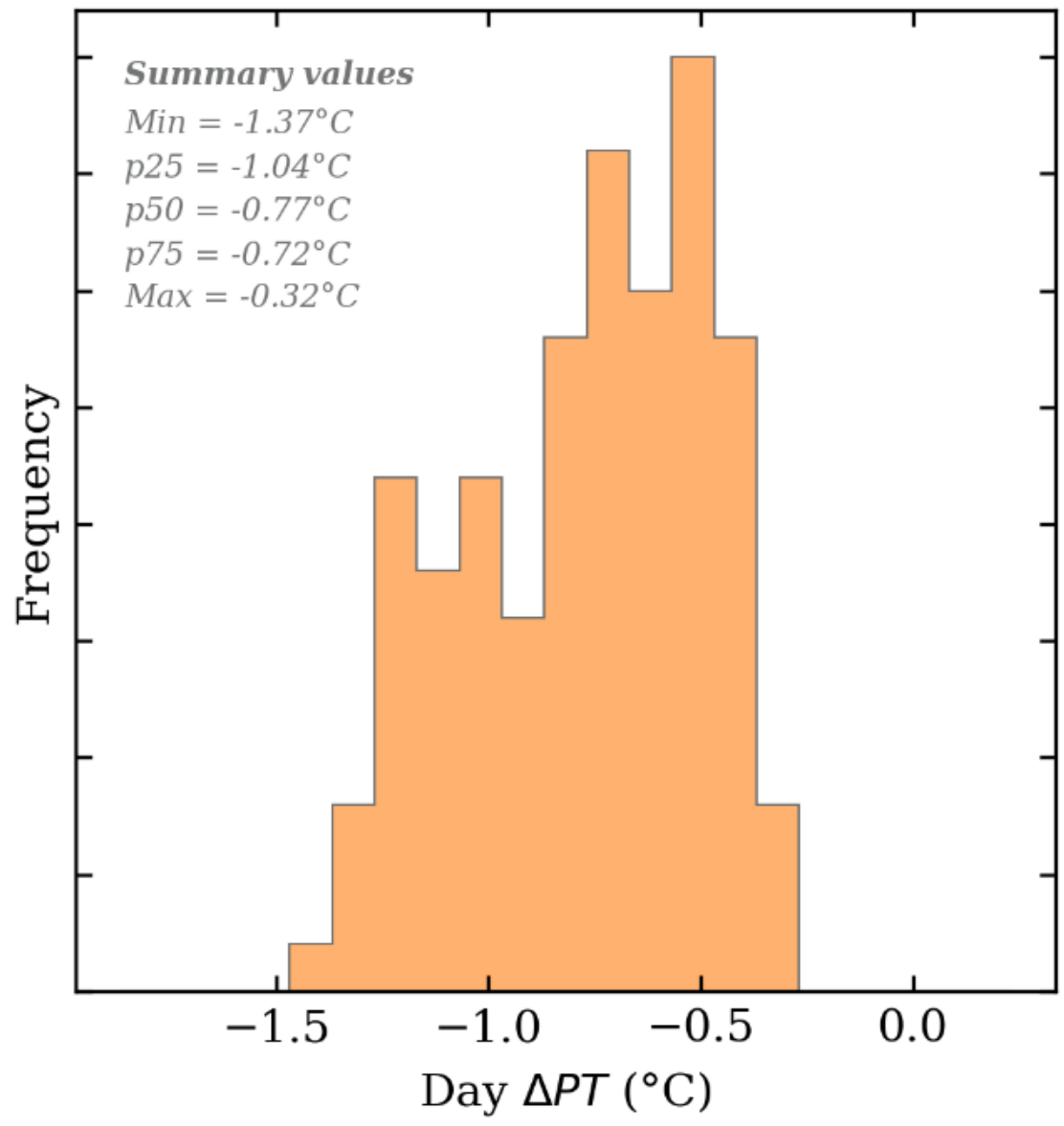

Supplementary Figure 6 Histogram of annual daytime ΔPT values for each region

Histogram of daytime ΔPT values for all studied regions (n=129), with summary statistics, including the minimum and maximum ΔPT, and the *p*25, *p*50, *p*75 values, weighted using canal area.

To investigate this, summary statistics were extracted for each region, including the average annual air temperature (K), humidity (%), air pressure (hPa), wind speed (m/s), cloud cover (%), canal width and depth (m) and degree of atmospheric shading by buildings (0-1). Ordinary least squares regression was performed for each attribute, which revealed a strong correlation between the median daytime ΔPT and cloud cover (R^2 = 0.67, p < 0.01) and with wind speed (R^2 = 0.51, p < 0.01), as shown in Supplementary Figure 7.

Increased daytime ΔPT (i.e., enhanced cooling) under scenarios of decreased cloud cover and increased wind speed is explained by an increased temperature difference between the water and the reference surface, which directly controls the net energy flux to the surrounding air. For cloud cover, this is accounted for by the differing specific heat capacities of the chosen materials, at ~1,020 J/kg K for asphalt (*19*) and ~4,186 J/kg K for water, with the temperature of the former increasing more rapidly when cloud cover is low and when total solar irradiance is increased.

The water–reference temperature difference is also enhanced when winds speeds are high, due the strong effect of wind on the rate of evaporation (*20*, *21*). An enhanced latent heat flux from the water to its surroundings with higher wind speeds is associated with evaporative cooling of the water body, and a greater temperature difference with respect to the reference material.

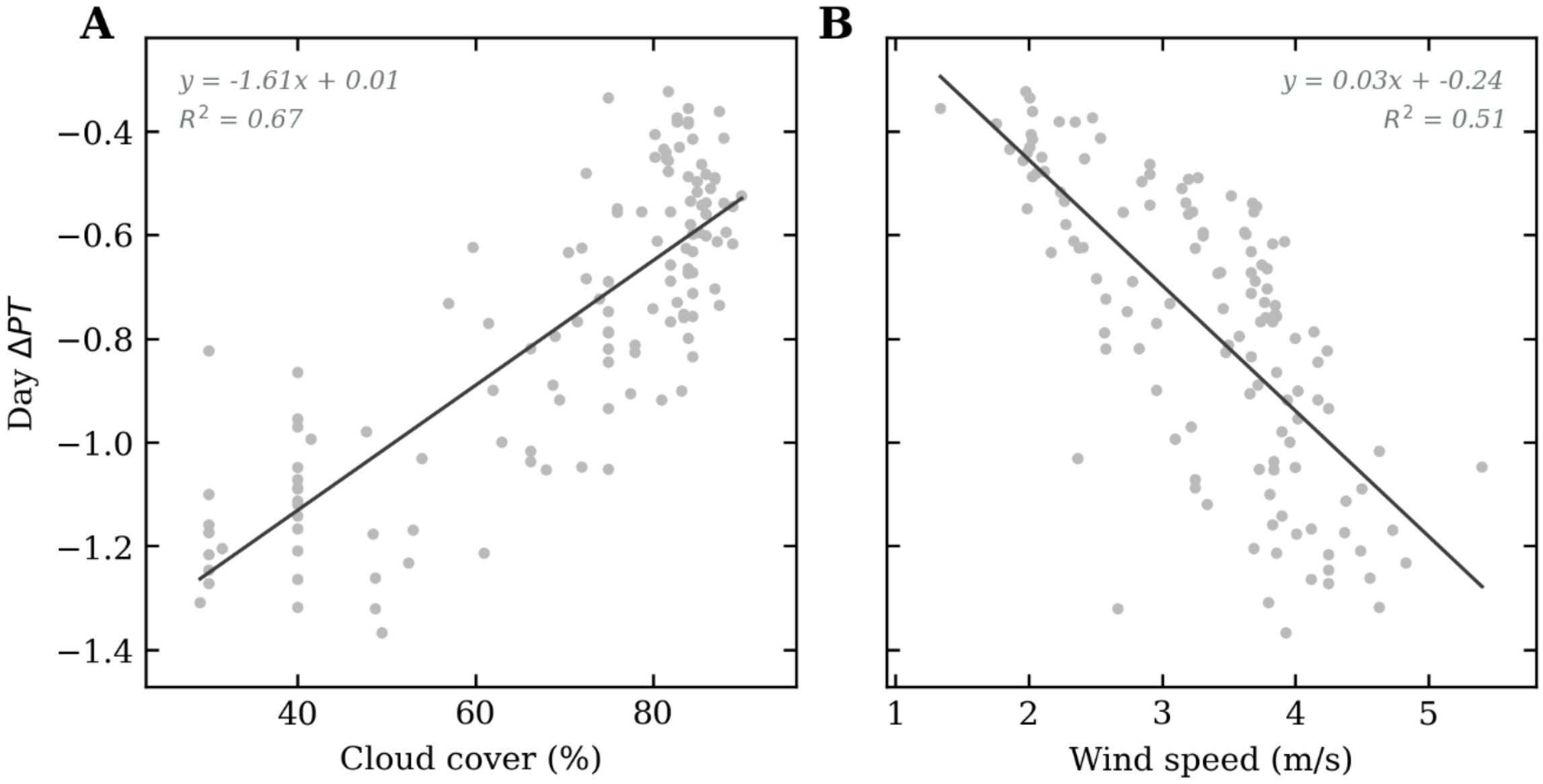

Supplementary Figure 7 Scatter plot of region attributes vs. median daytime ΔPT

(**A**) Average cloud cover (%) and (**B**) wind speed (m/s) for each region compared to median daytime ΔPT and the results of ordinary least squares regression.

Regional differences in median nighttime ΔPT warming are comparatively small, ranging from ~0.1 to 0.3°C, and are correlated with average humidity, as shown in Supplementary Figure 8. Increased nighttime ΔPT (i.e., enhanced warming) under scenarios of higher humidity is likely explained by reduced evaporative cooling and a supressed latent heat flux, resulting in higher water temperatures, and a greater temperature difference with respect to the reference surface.

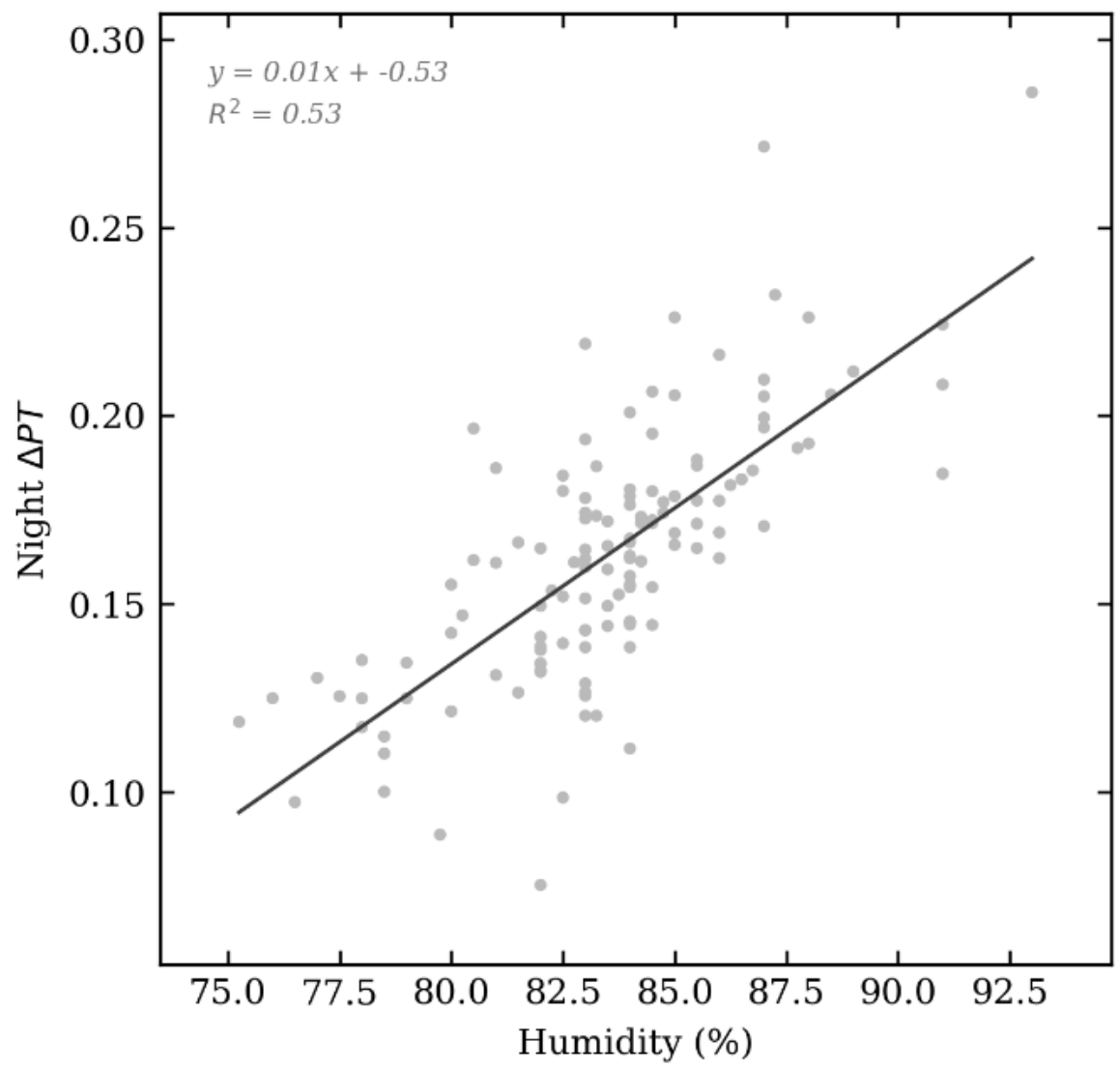

Supplementary Figure 8 Scatter plot of region attributes vs. median nighttime ΔPT

Average humidity (%) for each region compared to median nighttime ΔPT and the results of ordinary least squares regression.

Supplementary Discussion: Sensitivity testing of wet-bulb globe temperature

In our analysis we utilised a simplified version of wet-bulb globe temperature (WBGT), a thermal comfort index based on air temperature (°C) and humidity (%) (*36*, *37*). To investigate the impacts of canals, we calculated WGBT using the input meteorological data, and again with modified air temperature data, including canal effects (+ΔPT).

While this approach indicates that including ΔPT minimises the number of hours of "Moderate" (≥26°C), "High" (≥28°C) and "Extreme" heat risk (≥32°C) within the immediate vicinity of the canal, our approach is based on modified temperature alone, and does incorporate rising humidity due to evaporation (*22*), which has been shown to have an adverse effect on thermal comfort (*38*).

To investigate the sensitivity of our approach to changing humidity, WGBT (including ΔPT) was re-calculated for a range of plausible humidity values (+1% to +10%), with the results shown in Supplementary Figure 9. This approach indicates that the canal network would still reduce the number of hours of heat risk with moderate increases in humidity (approximately ≤10%) but would begin to negatively impact thermal comfort beyond this.

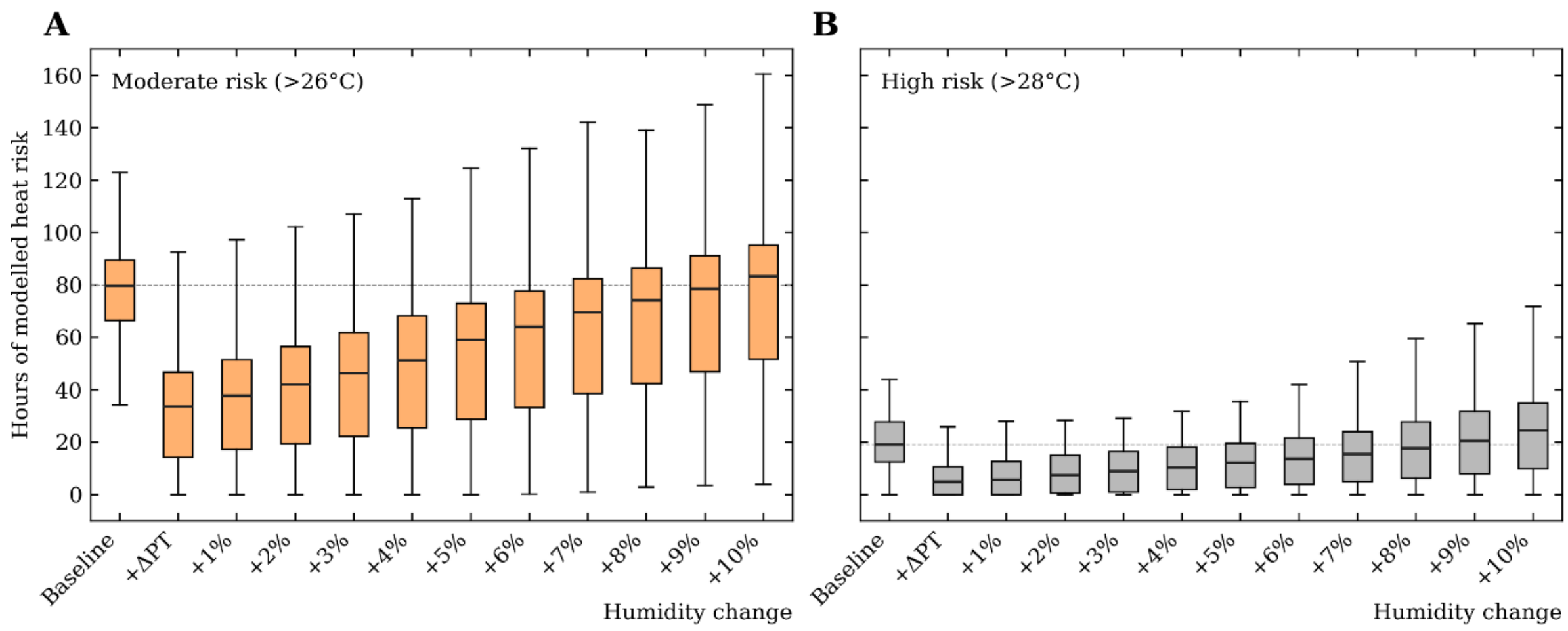

Supplementary Figure 9 Effects of ΔPT and changing humidity on modelled wet-bulb globe temperature

(A) Boxplots (Q1, Q2, Q3, where whiskers denote Q1–Q3 ±1.5 IQR) illustrating the number of hours of "moderate" heat risk (>26°C) and (**B**) "high" heat risk (>28°C) across the studied regions, using the input meteorological data ('Baseline'), incorporating canal effects (*+ΔPT*), and for increasing humidity values (+1 – 10%). Results for "extreme" heat risk (>32°C) are not plotted, given the comparatively small number of hours of risk across 2022 (*p*95 = 3.5 hours).

Supplementary Discussion: Canal network accessibility

To estimate the number of individuals who live within a ~15-minute walking distance of the canal network (~1 km), a commonly used metric in the field of urban planning (*22*, *23*), we utilised gridded population datasets derived from the latest census data, for 2021 for the UK (*24*) and 2022 for the Republic of Ireland (*25*). The canal network was buffered by 1 km and the population total within this area was calculated, resulting in ~5.9 million individuals in the UK and ~350,000 for Ireland.

To investigate how accessibility varied across the study area, the canal network was subset by region and the above methodology repeated. Region-specific population totals are reported in Supplementary Table 2, focusing on those with an accessible population >75,000. For each region, we calculated the total population within corresponding local authority areas to estimate the share of residents with access to the canal network (~15-minute walking distance). It should be noted that these estimates are sensitive to the calculation of the urban area population, which will vary as local authorities are included or excluded, or if more a spatially precise urban classification is used. Based on this approach, canals in Birmingham and London are accessible to the largest populations (~1.1 million individuals), and together account for ~37% of the total accessible population. When considered in the context of local authority populations, canals in Birmingham are serving the largest share (~45%), reflecting the size and distribution of the canal network, which stretches across much of the urban area. In contrast, canals are only easily accessible to a comparatively small share of the London population (~13%) due to the absence of canals from large parts of the city. Other regions, such as Leeds, Stoke-on-Trent, and Burnley and Nelson, also serve a large share of the local authority population (≥ 40%), reflecting the co-occurrence of canals and residential areas within each region.

It should be noted that the results above are based upon a Euclidean-based measure of accessibility, rather than a network-based approach (*26*), which would include the geometries of roads and paths, and access points to the canal network. While the former provides a useful first-order assessment of the accessibility of the canal network to urban populations (~6.2 million) and is appropriate given the resolution of the population grids (~1 km^2), a network analysis would be required for more detailed accessibility analysis.

Supplementary Table 2. Population within a 15-minute walking distance of the canal network (~1 km), subset by region. Population totals rounded to the nearest 1,000. For the designated UK regions, Government Statistical Service (GSS) codes are provided for corresponding local authorities. For Ireland, these are derived from the CSO Standard NUTS Regions and Local Authority (2019) Classification. To achieve the best match between designated regions and local authority areas, in some cases it was necessary to combine canal regions, as listed in the table footnotes.

Region	Population within 1 km	Local authority population	Proportion	Local authority IDs
Birmingham	1,159,000	2,550,000	0.45	E08000025, E08000027 – E08000031
London	1,138,000	8,814,000	0.13	E09000001 – E09000033
Manchester[1]	498,000	2,245,000	0.22	E08000002 – E08000009
Dublin	326,000	1,211,000	0.27	DC, SD, FC
Liverpool[2]	188,000	1,071,000	0.18	E08000012, E08000014, E08000015
Glasgow[3]	167,000	806,000	0.21	S12000039, S12000045, S12000049
Huddersfield[4]	114,000	434,000	0.26	E08000034
Burnley and Nelson[5]	105,000	191,000	0.55	E07000117, E07000122
Stoke-on-Trent	102,000	253,000	0.40	E06000021
Leeds	102,000	253,000	0.40	E08000035
Coventry	100,000	342,000	0.29	E08000026
Edinburgh	97,000	513,000	0.19	S12000036
Milton Keynes	84,000	284,000	0.30	E06000042

[1] including canals in Radcliffe, Rochdale, Littleborough, Saddleworth and Marple, [2] including canals in Maghull, [3] including canals in Kirkintilloch, [4] including canals in Mirfield and Dewsbury, [5] including canals in Barnoldswick

Supplementary Discussion: Short-term model performance

Based on comparison of modelled wT and measured wT for the annual time-series data (Figure 3), overall model performance is very good, with residuals commonly in the range ±1 − 2°C and Spearman's rank correlation coefficients of 0.57 – 0.81 ($p < 0.01$).

To investigate model performance over shorter timescales, and the degree to which modelled wT corresponds to measured wT over the course of a day, data were filtered to representative dates in March, June and September (Supplementary Figures 10 – 12). Relevant statistics were calculated, including absolute median residuals, to provide information on the average deviation of modelled wT from measured wT, and Spearman's rank correlation coefficients, to evaluate the strength and direction of association. These data provide useful insights into daily model performance, which largely reflects the broader trends observed when the annual time-series was used (Figure 3). For example, model performance is generally improved in June and September (Figures Supplementary Figures 11 – 12), with evidence of under-prediction in March (Supplementary Figure 10).

However, these examples provide only a snapshot of model performance, which might be expected to vary daily or hourly as a function of model parameterisation, the inclusion and correct representation of relevant environmental characteristics, and the quality of the input data. To investigate this further, we calculated absolute residuals for each hour of the day across the year (00:00:00 – 23:00:00, n = 24), with the results shown in Supplementary Figure 13. For the Leeds and Liverpool, Nottingham and Beeston and Huddersfield canals, model performance is consistent across the day (Supplementary Figure 13 A, B, E), with absolute median residuals of ~1 – 1.5°C. For the remaining canals there is evidence of some variability in model performance, with higher absolute residuals in the morning for the Shropshire Union and Coventry canals (Supplementary Figure 13 C, F), and in the afternoon for the Bridgewater and Taunton Canal (Supplementary Figure 13 D). This variability might reflect environmental characteristics or fluxes that it was not possible to include at the scale of analysis (e.g., shading by vegetation, heat flux due to bed conduction), or the accuracy of the input meteorological or environmental data. Overall, the consistent performance of many of the models (Supplementary Figure 13 A, B, E), as well as often small residuals (e.g., commonly <1°C for Supplementary Figure 13 F), indicates that important energy fluxes are being represented correctly in the energy balance model.

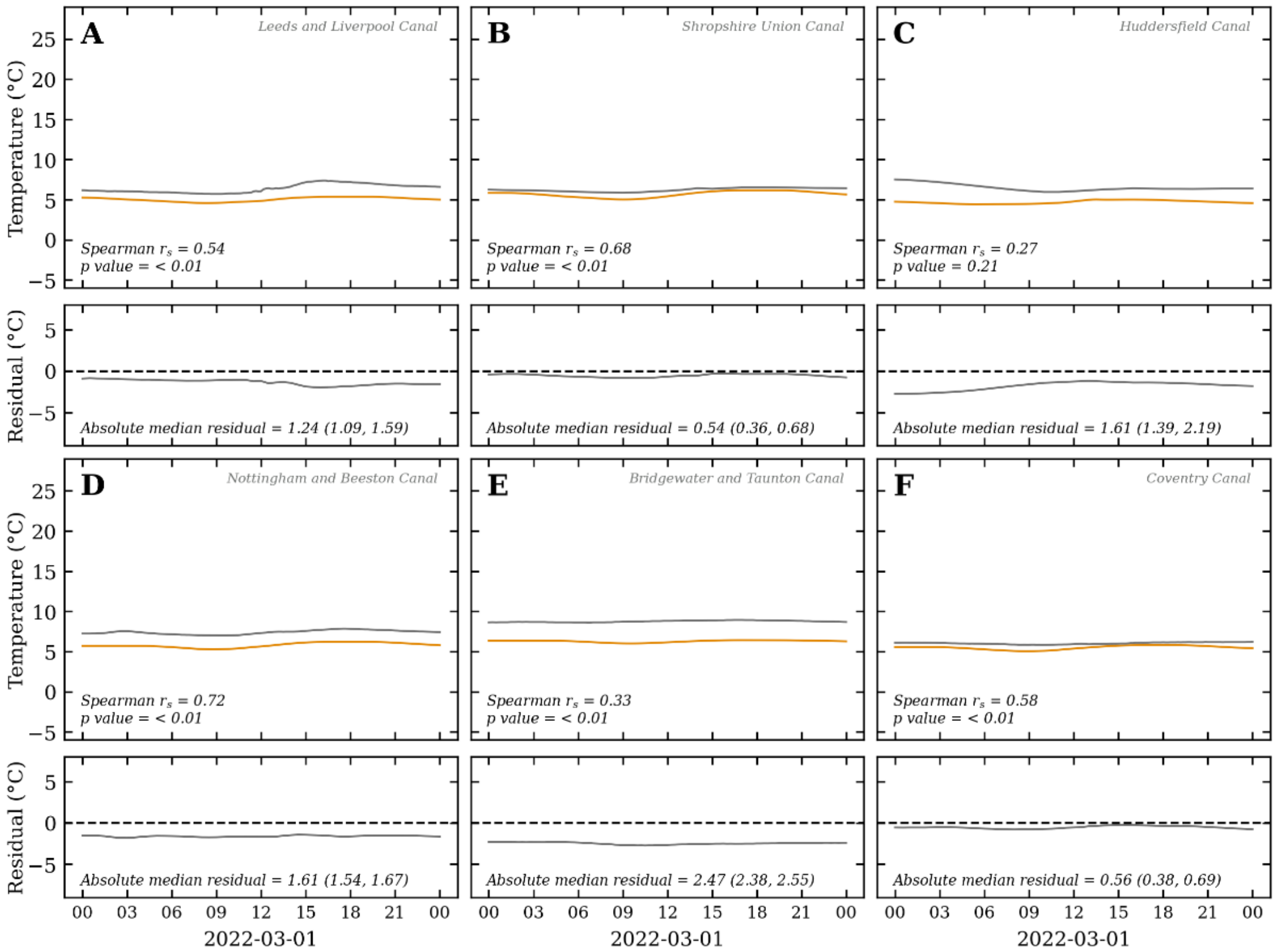

Supplementary Figure 10 Comparison of measured vs. modelled *wT* for 1st March 2022

Comparison of modelled wT at 40 – 60 cm depth within the water column (orange lines) with measured wT for the same depth (grey lines), plus residuals (absolute median, *p*25, *p*75). Measured data were provided by the Canal and River Trust and were available for the Leeds and Liverpool Canal (**A**), the Shropshire Union Canal (**B**), the Huddersfield Canal (**C**), the Nottingham and Beeston Canal (**D**), the Bridgwater and Taunton Canal (**E**), and the Coventry Canal (**F**). Water temperature was measured at 10 minute (A, D, F), 15 minute (E), or hourly frequency (B, C). Spearman's rank correlation coefficient (r_s, *p* value) was calculated based on stationary time series, produced using the differences between consecutive observations.

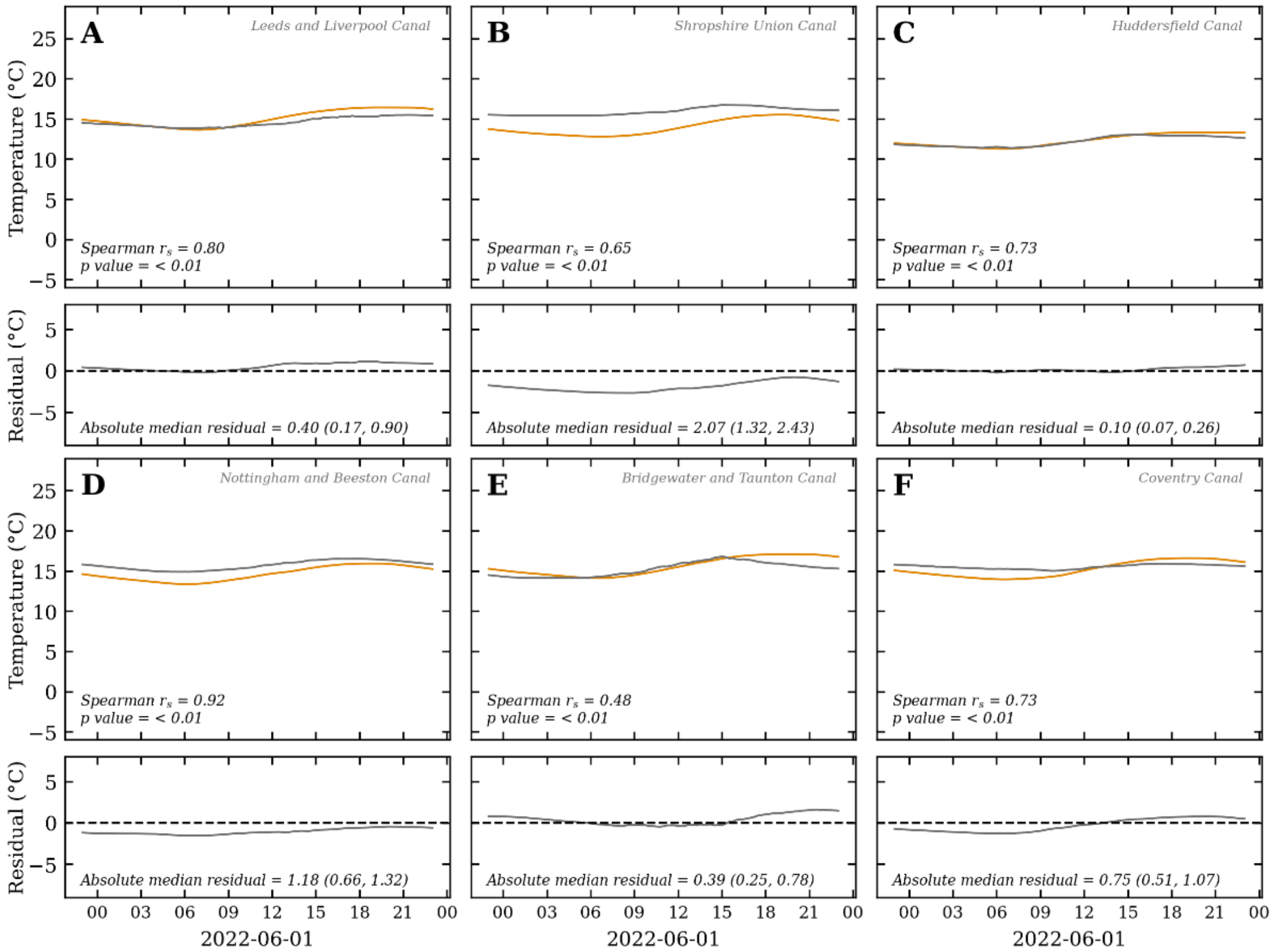

Supplementary Figure 11 Comparison of measured vs. modelled *wT* for 1st June 2022

Comparison of modelled wT at 40 – 60 cm depth within the water column (orange lines) with measured wT for the same depth (grey lines), plus residuals (absolute median, *p*25, *p*75). Measured data were provided by the Canal and River Trust and were available for the Leeds and Liverpool Canal (**A**), the Shropshire Union Canal (**B**), the Huddersfield Canal (**C**), the Nottingham and Beeston Canal (**D**), the Bridgwater and Taunton Canal (**E**), and the Coventry Canal (**F**). Water temperature was measured at 10 minute (A, D, F), 15 minute (E), or hourly frequency (B, C). Spearman's rank correlation coefficient (r_s, *p* value) was calculated based on stationary time series, produced using the differences between consecutive observations.

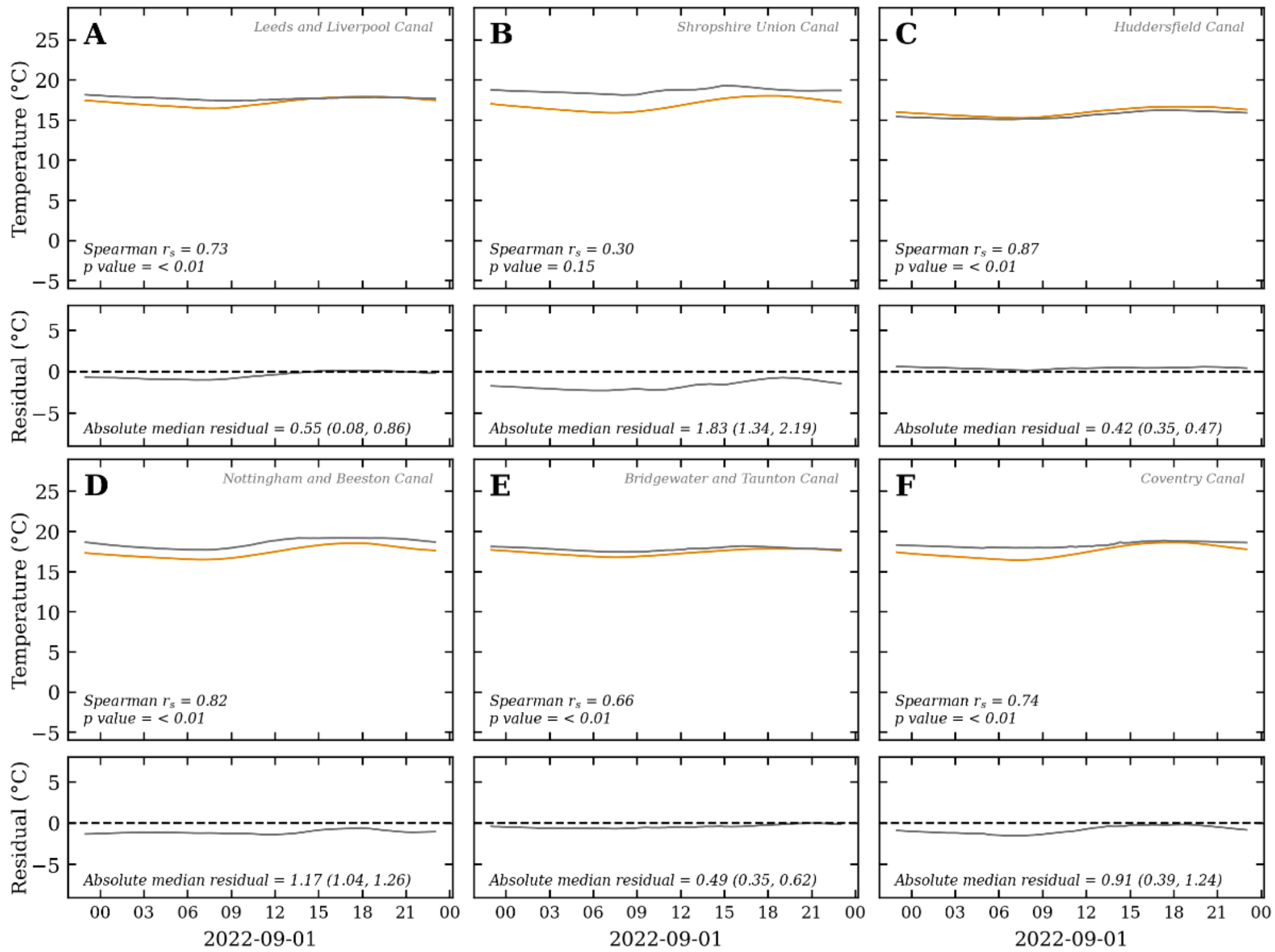

Supplementary Figure 12 Comparison of measured vs. modelled *wT* for 1st September 2022

Comparison of modelled wT at 40 – 60 cm depth within the water column (orange lines) with measured wT for the same depth (grey lines), plus residuals (absolute median, *p*25, *p*75). Measured data were provided by the Canal and River Trust and were available for the Leeds and Liverpool Canal (**A**), the Shropshire Union Canal (**B**), the Huddersfield Canal (**C**), the Nottingham and Beeston Canal (**D**), the Bridgwater and Taunton Canal (**E**), and the Coventry Canal (**F**). Water temperature was measured at 10 minute (A, D, F), 15 minute (E), or hourly frequency (B, C). Spearman's rank correlation coefficient (r_s, *p* value) was calculated based on stationary time series, produced using the differences between consecutive observations.

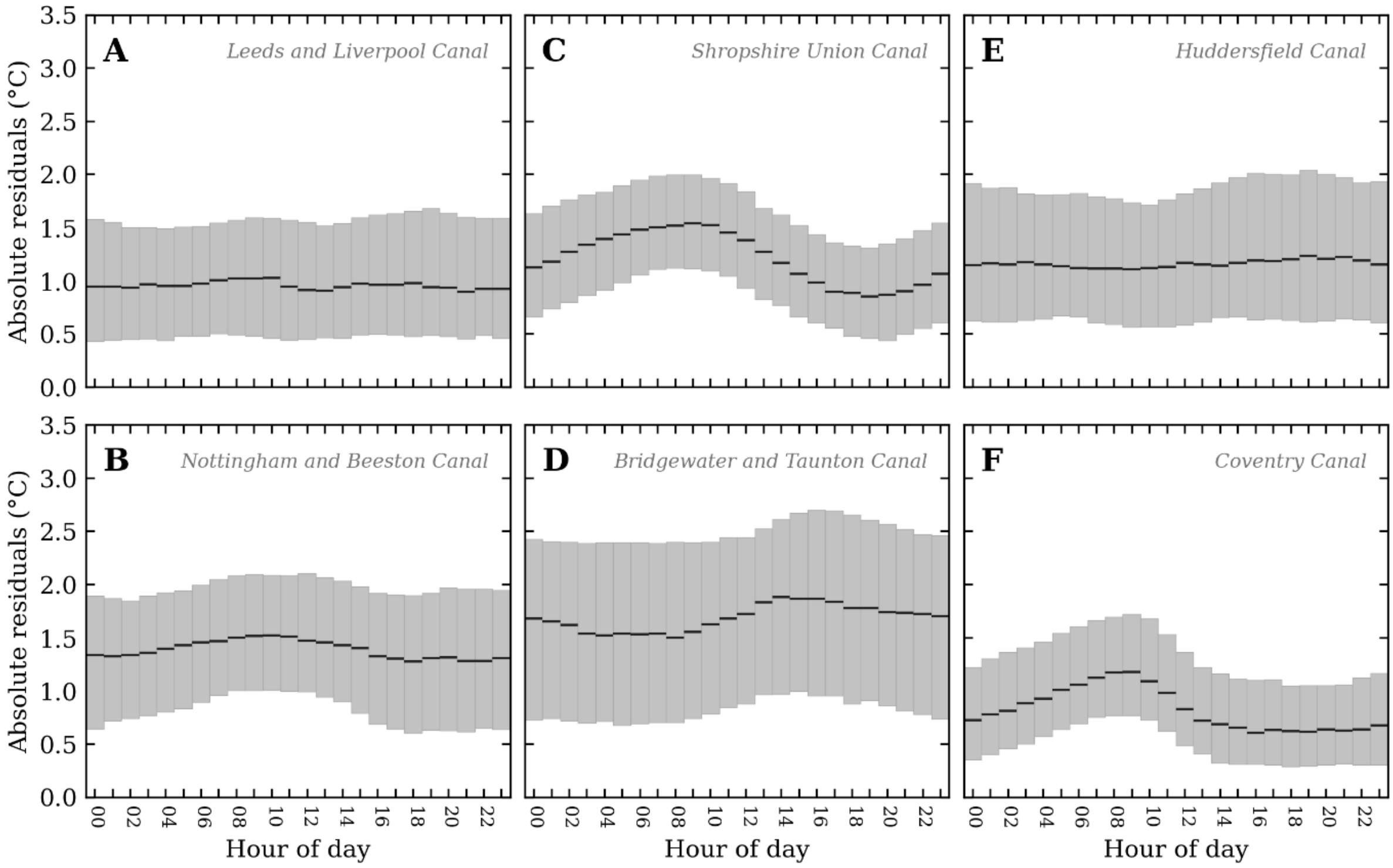

Supplementary Figure 13 Hourly absolute model residuals for measured vs. modelled *wT*

Absolute residuals for each hour of the day, showing the median (black line) and *p*25 – *p*75 (grey shading), between modelled wT at 40 – 60 cm depth within the water column with measured wT for the same depth absolute median, *p*25, *p*75). Measured data were provided by the Canal and River Trust and were available for the Leeds and Liverpool Canal (**A**), the Shropshire Union Canal (**B**), the Huddersfield Canal (**C**), the Nottingham and Beeston Canal (**D**), the Bridgwater and Taunton Canal (**E**), and the Coventry Canal (**F**). Water temperature was measured at 10 minute (A, D, F), 15 minute (E), or hourly frequency (B, C).

Supplementary References

1. B. W. Webb, Y. Zhang, Spatial and Seasonal Variability in the Components of the River Heat Budget. *Hydrol. Process.* **11**, 79–101 (1997).

2. P. Bretagnon, G. Francou, Planetary theories in rectangular and spherical variables-VSOP 87 solutions. *Astron. Astrophys. ISSN 0004-6361 Vol 202 No 1-2 Aug 1988 P 309-315* **202**, 309–315 (1988).

3. B. Stafford, pysolar, Zenodo (2021); https://doi.org/10.5281/zenodo.8184418.

4. M. G. Nevins, J. N. Apell, Emerging investigator series: quantifying the impact of cloud cover on solar irradiance and environmental photodegradation. *Environ. Sci. Process. Impacts* **23**, 1884–1892 (2021).

5. M. A. Goforth, G. W. Gilchrist, J. D. Sirianni, “Cloud effects on thermal downwelling sky radiance” in *Thermosense XXIV* (SPIE, 2002)vol. 4710, pp. 203–213.

6. R. D. Moore, J. A. Leach, Predicting Latent and Sensible Heat Fluxes in Stream Temperature Models: Current Challenges and Potential Solutions. *Water Resour. Res.* **57**, e2020WR028712 (2021).

7. I. E. Gordon, L. S. Rothman, R. J. Hargreaves, R. Hashemi, E. V. Karlovets, F. M. Skinner, E. K. Conway, C. Hill, R. V. Kochanov, Y. Tan, P. Wcisło, A. A. Finenko, K. Nelson, P. F. Bernath, M. Birk, V. Boudon, A. Campargue, K. V. Chance, A. Coustenis, B. J. Drouin, J. –M. Flaud, R. R. Gamache, J. T. Hodges, D. Jacquemart, E. J. Mlawer, A. V. Nikitin, V. I. Perevalov, M. Rotger, J. Tennyson, G. C. Toon, H. Tran, V. G. Tyuterev, E. M. Adkins, A. Baker, A. Barbe, E. Canè, A. G. Császár, A. Dudaryonok, O. Egorov, A. J. Fleisher, H. Fleurbaey, A. Foltynowicz, T. Furtenbacher, J. J. Harrison, J. –M. Hartmann, V. –M. Horneman, X. Huang, T. Karman, J. Karns, S. Kassi, I. Kleiner, V. Kofman, F. Kwabia–Tchana, N. N. Lavrentieva, T. J. Lee, D. A. Long, A. A. Lukashevskaya, O. M. Lyulin, V. Yu. Makhnev, W. Matt, S. T. Massie, M. Melosso, S. N. Mikhailenko, D. Mondelain, H. S. P. Müller, O. V. Naumenko, A. Perrin, O. L. Polyansky, E. Raddaoui, P. L. Raston, Z. D. Reed, M. Rey, C. Richard, R. Tóbiás, I. Sadiek, D. W. Schwenke, E. Starikova, K. Sung, F. Tamassia, S. A. Tashkun, J. Vander Auwera, I. A. Vasilenko, A. A. Vigasin, G. L. Villanueva, B. Vispoel, G. Wagner, A. Yachmenev, S. N. Yurchenko, The HITRAN2020 molecular spectroscopic database. *J. Quant. Spectrosc. Radiat. Transf.* **277**, 107949 (2022).

8. C. Jacobs, L. Klok, M. Bruse, J. Cortesão, S. Lenzholzer, J. Kluck, Are urban water bodies really cooling? *Urban Clim.* **32**, 100607 (2020).

9. D. M. Hannah, G. Garner, River water temperature in the United Kingdom: Changes over the 20th century and possible changes over the 21st century. *Prog. Phys. Geogr. Earth Environ.* **39**, 68–92 (2015).

10. R. Sun, L. Chen, How can urban water bodies be designed for climate adaptation? *Landsc. Urban Plan.* **105**, 27–33 (2012).

11. Y. Zhou, Y. Lu, X. Zhou, J. An, D. Yan, Numerical study on the coupling effect of river attributes and riverside building forms on the urban microclimate: A case study in Nanjing, China. *Sustain. Cities Soc.* **107**, 105459 (2024).

12. E. A. Hathway, S. Sharples, The interaction of rivers and urban form in mitigating the Urban Heat Island effect: A UK case study. *Build. Environ.* **58**, 14–22 (2012).

13. S. Murakawa, T. Sekine, K. Narita, D. Nishina, Study of the effects of a river on the thermal environment in an urban area. *Energy Build.* **16**, 993–1001 (1991).

14. C. Y. Park, D. K. Lee, T. Asawa, A. Murakami, H. G. Kim, M. K. Lee, H. S. Lee, Influence of urban form on the cooling effect of a small urban river. *Landsc. Urban Plan.* **183**, 26–35 (2019).

15. H. Du, X. Song, H. Jiang, Z. Kan, Z. Wang, Y. Cai, Research on the cooling island effects of water body: A case study of Shanghai, China. *Ecol. Indic.* **67**, 31–38 (2016).

16. H. Saaroni, B. Ziv, The impact of a small lake on heat stress in a Mediterranean urban park: the case of Tel Aviv, Israel. *Int. J. Biometeorol.* **47**, 156–165 (2003).

17. L. Fricke, R. Legg, N. Kabisch, Impact of blue spaces on the urban microclimate in different climate zones, daytimes and seasons – A systematic review. *Urban For. Urban Green.* **101**, 128528 (2024).

18. A. M. Broadbent, A. M. Coutts, N. J. Tapper, M. Demuzere, J. Beringer, The microscale cooling effects of water sensitive urban design and irrigation in a suburban environment. *Theor. Appl. Climatol.* **134**, 1–23 (2018).

19. J. Byzyka, M. Rahman, D. A. Chamberlain, A laboratory investigation on thermal properties of virgin and aged asphalt mixture. *Constr. Build. Mater.* **305**, 124757 (2021).

20. H. L. Penman, Natural evaporation from open water, bare soil and grass. *Proc. R. Soc. Lond. Ser. Math. Phys. Sci.* **193**, 120–145 (1948).

21. J. D. Valiantzas, Simplified versions for the Penman evaporation equation using routine weather data. *J. Hydrol.* **331**, 690–702 (2006).

22. E. Willberg, C. Fink, T. Toivonen, The 15-minute city for all? – Measuring individual and temporal variations in walking accessibility. *J. Transp. Geogr.* **106**, 103521 (2023).

23. M. Weng, N. Ding, J. Li, X. Jin, H. Xiao, Z. He, S. Su, The 15-minute walkable neighborhoods: Measurement, social inequalities and implications for building healthy communities in urban China. *J. Transp. Health* **13**, 259–273 (2019).

24. E. Carnell, S. J. Tomlinson, S. Reis, UK gridded population at 1 km resolution for 2021 based on Census 2021/2022 and Land Cover Map 2021 (2025). https://doi.org/10.5285/7beefde9-c520-4ddf-897a-0167e8918595.

25. Central Statistics Office, Census 2022 Grid dataset, (2023).

26. G. Higgs, R. Fry, M. Langford, Investigating the Implications of Using Alternative GIS-Based Techniques to Measure Accessibility to Green Space. *Environ. Plan. B Plan. Des.* **39**, 326–343 (2012).
