# Supplementary material for: A national-scale assessment of the impact of canals on urban temperatures
Source: Nat Commun. 2026 Aug 10;17:7849. doi: 10.1038/s41467-026-75731-0 (PMC13458440; doi:10.1038/s41467-026-75731-0)
Supplement: Supplementary file 2 [file 41467_2026_75731_MOESM2_ESM.pdf]

nature portfolio

Corresponding author(s): Matthew Tomkins

Last updated by author(s): May 6, 2026

Reporting Summary

Nature Portfolio wishes to improve the reproducibility of the work that we publish. This form provides structure for consistency and transparency in reporting. For further information on Nature Portfolio policies, see our Editorial Policies and the Editorial Policy Checklist.

Statistics

For all statistical analyses, confirm that the following items are present in the figure legend, table legend, main text, or Methods section.

n/a	Confirmed	
☐	☒	The exact sample size (*n*) for each experimental group/condition, given as a discrete number and unit of measurement
☒	☐	A statement on whether measurements were taken from distinct samples or whether the same sample was measured repeatedly
☐	☒	The statistical test(s) used AND whether they are one- or two-sided<br>*Only common tests should be described solely by name; describe more complex techniques in the Methods section.*
☐	☒	A description of all covariates tested
☐	☒	A description of any assumptions or corrections, such as tests of normality and adjustment for multiple comparisons
☐	☒	A full description of the statistical parameters including central tendency (e.g. means) or other basic estimates (e.g. regression coefficient) AND variation (e.g. standard deviation) or associated estimates of uncertainty (e.g. confidence intervals)
☐	☒	For null hypothesis testing, the test statistic (e.g. *F*, *t*, *r*) with confidence intervals, effect sizes, degrees of freedom and *P* value noted<br>*Give P values as exact values whenever suitable.*
☒	☐	For Bayesian analysis, information on the choice of priors and Markov chain Monte Carlo settings
☒	☐	For hierarchical and complex designs, identification of the appropriate level for tests and full reporting of outcomes
☐	☒	Estimates of effect sizes (e.g. Cohen's *d*, Pearson's *r*), indicating how they were calculated

Our web collection on statistics for biologists contains articles on many of the points above.

Software and code

Policy information about availability of computer code

Data collection	Model code, key functions and parameters are available on Git: https://github.com/matt-tomkins/national-canal-cooling.
Data analysis	Code for generating results and plots (analysis.py) are also available on Git: https://github.com/matt-tomkins/national-canal-cooling.

For manuscripts utilizing custom algorithms or software that are central to the research but not yet described in published literature, software must be made available to editors and reviewers. We strongly encourage code deposition in a community repository (e.g. GitHub). See the Nature Portfolio guidelines for submitting code & software for further information.

Data

Policy information about availability of data

All manuscripts must include a data availability statement. This statement should provide the following information, where applicable:

- Accession codes, unique identifiers, or web links for publicly available datasets
- A description of any restrictions on data availability
- For clinical datasets or third party data, please ensure that the statement adheres to our policy

Our models are run using proprietary data which is not possible to provide in full. This includes weather data (OpenWeather), canal and building geometries (Ordnance Survey), land cover data (UK Centre for Ecology and Hydrology), and canal characteristics (Canal and River Trust). While example datasets have been provided in the code repository to allow for model testing, users would need to utilise open-source alternatives to apply the models more widely e.g., OpenStreetMap.

Research involving human participants, their data, or biological material

Policy information about studies with human participants or human data. See also policy information about sex, gender (identity/presentation), and sexual orientation and race, ethnicity and racism.

Reporting on sex and gender	Sex- and gender-based analysis not relevant for this study.
Reporting on race, ethnicity, or other socially relevant groupings	Race, ethnicity, or other socially relevant groupings not included in the analysis.
Population characteristics	For assessing the accessibility of the canal network, we calculated the total population within a c. 15-minute walking distance (c. 1 km), as discussed in the manuscript and outlined in the Supplementary Information. There were no research participants, and we did not subset this population by demographic group.
Recruitment	No participants were recruited.
Ethics oversight	Human participants and human data were not analysed.

Note that full information on the approval of the study protocol must also be provided in the manuscript.

Field-specific reporting

Please select the one below that is the best fit for your research. If you are not sure, read the appropriate sections before making your selection.

☐ Life sciences ☐ Behavioural & social sciences ☒ Ecological, evolutionary & environmental sciences

For a reference copy of the document with all sections, see nature.com/documents/nr-reporting-summary-flat.pdf

Ecological, evolutionary & environmental sciences study design

All studies must disclose on these points even when the disclosure is negative.

Study description	In this study, we modelled the thermal effects of canals on the surrounding environment, focusing on the canals of Great Britain and Ireland. Energy fluxes associated with changing water energy balance through time were compared to those of a reference surface to estimate the potential change in air temperature.
Research sample	No sampling was conducted. The model was run using weather data (OpenWeather), canal and building geometries (Ordnance Survey, OpenStreetMap), land cover data (UK Centre for Ecology and Hydrology), and canal characteristics (Canal and River Trust).
Sampling strategy	Our research provides a national-scale perspective on the thermal effects of canals. In turn, our sample comprised all canals in Great Britain and Ireland, using canal geometries from the Ordnance Survey and OpenStreetMap, filtered to urban areas, and with canal characteristics obtained from the Canal and River Trust.
Data collection	No primary data collection. Secondary data obtained from OpenWeather (weather), the Ordnance Survey and OpenStreetMap (canal and building geometries), the UK Centre for Ecology and Hydrology (land cover) and the Canal and River Trust (canal characteristics).
Timing and spatial scale	The model was applied to the urban canals of Great Britain and Ireland (national-scale), with the model run for the entirety of 2022 (15-minute model resolution).
Data exclusions	We excluded canals outside of urban areas based on land cover data.
Reproducibility	Full implementation available on Git: https://github.com/matt-tomkins/national-canal-cooling.
Randomization	The model is deterministic, so no randomisation utilized.
Blinding	Our study was not a randomized controlled trial, so no blinding required.

Did the study involve field work? ☐ Yes ☒ No

Reporting for specific materials, systems and methods

We require information from authors about some types of materials, experimental systems and methods used in many studies. Here, indicate whether each material, system or method listed is relevant to your study. If you are not sure if a list item applies to your research, read the appropriate section before selecting a response.

Materials & experimental systems

n/a	Involved in the study
☒	☐ Antibodies
☒	☐ Eukaryotic cell lines
☒	☐ Palaeontology and archaeology
☒	☐ Animals and other organisms
☒	☐ Clinical data
☒	☐ Dual use research of concern
☒	☐ Plants

Methods

n/a	Involved in the study
☒	☐ ChIP-seq
☒	☐ Flow cytometry
☒	☐ MRI-based neuroimaging

Plants

Seed stocks	No seed stocks used.
Novel plant genotypes	No novel plant genotypes were used
Authentication	No seed stocks used, so no authentication required.
