# Supplementary material for: A national-scale assessment of the impact of canals on urban temperatures
Source: Nat Commun. 2026 Aug 10;17:7849. doi: 10.1038/s41467-026-75731-0 (PMC13458440; doi:10.1038/s41467-026-75731-0)
Supplement: Supplementary file 3 [file 41467_2026_75731_MOESM3_ESM.pdf]

nature portfolio

Peer Review File

A national-scale assessment of the impact of canals on urban temperatures

Corresponding Author: Dr Matt Tomkins

This file contains all reviewer reports in order by version, followed by all author rebuttals in order by version.

Version 0:

Reviewer comments:

Reviewer #1

(Remarks to the Author)
This is an interesting topic and worthy of study but I feel the paper needs to be expanded to improve the readability and quality.

Due to the layout of the document with the methodology at the end we are presented with a very brief introduction and then some results that only mention a 'model' without any details. Overall the introduction lacks sufficient detail to prepare the reader for the results - we need a short paragraph at the end of the intro to describe the model and what it does, along with a rationale for this type of model versus say a full microclimate simulation.

The data in fig 3 is I assume over a year - the axes are poorly labelled. We have no information about the frequency of measurement. We also have no information about how well the modelled data maps the monitored data over the course of a day. Does the energy balance model accurately predict surface water temperatures over the course of a day? Details are lacking in the method but I assume the water is treated as fully mixed when in reality a static waterbody like a canal is likely stratified. As such what confidence do we have that the evaporated, convected and radiated heat from the water surface is accurate?

Of greatest concern are the statements regarding improvements to thermal comfort. The results presented are all in terms of temperature improvement only. There is no consideration of humidity or radiant temperatures which are also significant aspects of thermal comfort. The use of asphalt as the reference material also serves to improve the stated cooling potential.

As a minimum I would like to see a greater justification for the use of asphalt over say concrete or bare soil as the reference material, along with details of how this material was incorporated into the model (depth, conductivity, heat capacity and emissivity). I also require statements regarding thermal comfort and heat stress reduction to be reframed to air temperature improvements.

The methodology lacks detail - the basic heat balance equation is introduced but there is no detail on how the constituents such as Qe, Qh or Qr are calculated.

Overall this is a good paper on a worthy topic which will contribute to the field but we require significantly more information within the paper itself so there reader can make informed decisions.

(Remarks on code availability)

Reviewer #2

(Remarks to the Author)
The influence of urban blue spaces on microclimate regulation is comparatively underrepresented in the literature compared to the extensive work on urban green infrastructure. This gap is particularly evident in narrow urban waterways such as canals, which have received far less attention than lakes. Furthermore, modeling approaches are still less common than studies based on remote sensing or field measurements, but they offer various advantages, such as the ability to capture day

and night assessments. Against this background, the present study makes a valuable contribution to this field of research. The focus on canal systems in urban areas and the inclusion of WBGT as an indicator of thermal comfort for pedestrians further increase the relevance and practical significance of the study.
A notable strength of the study is the validation of the modeled water temperatures using measurements from multiple monitoring sites, which increases confidence in the modeling approach. Another important strength is the simulation of a complete annual cycle, which allows for the assessment of the seasonal variation as well as the nocturnal thermal effects of canals – an aspect that is often neglected, as many remote sensing-based studies primarily capture daytime conditions.

Overall, the methodology is well-founded and described in sufficient detail to ensure reproducibility. The reported results appear plausible and are consistent with the values reported in the literature. The conclusions are generally supported by the modeling results presented.

The following points may help improve clarity and interpretation:
• The section discussing the impact on thermal comfort based on the WBGT index contains direct modeling results and might be better placed in the "Results" section.

• The discussion should emphasize that the input data on land use are greatly simplified and that the absence of vegetation probably intensifies thermal contrasts and may lead to an overestimation of cooling values and heat stress (WBGT). This simplification does not invalidate the results but should be more clearly framed as a modelling assumption and limitation.

• The discussion could further highlight the relevance of this modeling approach for urban planning applications. For practical implementation, future model development should incorporate more detailed representations of land use, including low vegetation and tree cover.

(Remarks on code availability)

Reviewer #3

(Remarks to the Author)
This manuscript conducts a national-scale quantitative assessment of the cooling effects of urban canal sections across Great Britain and Ireland by constructing a physical energy balance model. The research is innovative in its integration of physical modeling with temporal and seasonal analysis, providing a valuable quantitative reference for the microclimate effects of urban canals. However, the rationality of the model's simplification in the transition from water temperature simulation to air temperature effect needs further refinement. The current model architecture may significantly overestimate the cooling benefits of canals, and supplementary validation experiments for key physical processes are required.
(1) The literature review on the cooling effects of urban blue spaces is insufficient, with the cited references being as old as 2021. Several relevant articles on the cooling effects of rivers have been published in recent years, and the statements and references regarding certain controversies should be updated accordingly, especially since the author already addressed a recent survey (Fricke et al., 2024) in the discussion.
References
[1] Fricke L, Legg R, Kabisch N. Impact of blue spaces on the urban microclimate in different climate zones, daytimes and seasons–A systematic review [J]. Urban Forestry & Urban Greening, 2024, 101: 128528.
[2] Wang Y, Lai X, Liu Y, et al. A Review of Research on the Impact of Urban Blue-Green Spaces on Urban Cooling Effect[C]. International Conference on Energy and Environmental Science. Cham: Springer Nature Switzerland, 2025: 898-912.
[3] Zhang L, Meng Q, Yao S, et al. How to optimize urban blue space to maximize its cooling benefits? A case study in megacity of Beijing[J]. Building and Environment, 2025, 270: 112502.
[4] Das M, Das A, Rajjack A, et al. Understanding the seasonal pattern and factors affecting cooling effect of blue spaces: a case from a mega metropolitan area (India)[J]. Sustainable Cities and Society, 2025: 106656.
(2) The core design of the model relies on an energy balance equation, validating the model by comparing measured and simulated water temperatures through a series of idealized simplifications regarding underlying surfaces, meteorology, and urban morphology. Is using a "single asphalt reference surface" an oversimplification of the energy model design? It remains unclear whether the effects would change if the surrounding reference surfaces were grass or trees, or if the 3D building density of the surrounding city affects the cooling distance and intensity. Detailed descriptions of different underlying surface compositions and supplementary experiments are missing.
(3) The abstract of this paper claims to have developed a novel energy balance model. Compared to existing research on energy equation models over the years, what are the specific breakthroughs and contributions of this study? Furthermore, are there any significant differences in the energy balance equations constructed when targeting canals versus lakes for calculating cooling effects?
[1] Mitchell V G, Cleugh H A, Grimmond C S B, et al. Linking urban water balance and energy balance models to analyse urban design options[J]. Hydrological Processes: An International Journal, 2008, 22(16): 2891-2900.
[2] Henderson-Sellers B. Calculating the surface energy balance for lake and reservoir modeling: A review[J]. Reviews of geophysics, 1986, 24(3): 625-649.
[3] House-Peters L A, Chang H. Modeling the impact of land use and climate change on neighborhood-scale evaporation and nighttime cooling: A surface energy balance approach[J]. Landscape and urban planning, 2011, 103(2): 139-155.
[4] Rupper S, Roe G. Glacier changes and regional climate: a mass and energy balance approach[J]. Journal of Climate, 2008, 21(20): 5384-5401.

(4) In the results, the interpretation of the canal's morphological parameters primarily uses "area" as the main variable. The narrow and elongated state of canals, along with other morphological parameters, are not elaborated upon in detail. The study offers limited insights into how water body types, different scales, and various shapes of canals contribute to the explanatory power of the cooling effect.
(5) In the discussion, the author clearly explains the simplification of the energy equation and the humidity-rising effect caused by evaporation, which is commendable. However, many processes and mechanistic effects have not been fully accounted for. Calculating △PT and the cooling-effect-corrected WBGT through extreme simplification is problematic, as these are precisely the elements that need accurate measurement in current research and findings.
(6) Supplementary Material S1 presents a “full energy equation” but omits latent heat flux. Because the study spans full seasonal cycles, including winter conditions where water surfaces may freeze, further adjustments to the energy equation parameters are necessary to account for these scenarios.
(7) The empirical fit in Panel A of Supplementary Figure S4 looks suspicious. The distribution is highly skewed and appears to rely on only the first and last data points, rather than a proper regression across the full dataset.
(8) Regarding Fig. S7 (Scatter plot of region attributes vs. median daytime △PT), why is the nighttime situation not supplemented?
(9) Could Section S5 be expanded to include city-level breakdowns of population size and the share of residents living within 1 km of canals? This would make it clearer how many people actually benefit from the cooling effect and how these benefits are distributed across the study area.

(Remarks on code availability)
I have carefully reviewed the code and found no obvious errors.

Version 1:

Reviewer comments:

Reviewer #1

(Remarks to the Author)
Thank you for your detailed responses to my comments, I am satisfied with the changes to the paper and supplementary material.

(Remarks on code availability)
I am not qualified to review the code.

Reviewer #2

(Remarks to the Author)
I appreciate the authors for their thorough revision of the manuscript and for addressing my comments clearly and satisfactorily.

In my view, the comments raised in my review have been adequately addressed, and I have no further comments.

(Remarks on code availability)

Reviewer #3

(Remarks to the Author)
The authors have responded well to my queries and suggestions, and have made adequate and corresponding improvements. I have no further questions.

(Remarks on code availability)

Open Access This Peer Review File is licensed under a Creative Commons Attribution 4.0 International License, which permits use, sharing, adaptation, distribution and reproduction in any medium or format, as long as you give appropriate credit to the original author(s) and the source, provide a link to the Creative Commons license, and indicate if changes were made.
In cases where reviewers are anonymous, credit should be given to 'Anonymous Referee' and the source.
The images or other third party material in this Peer Review File are included in the article's Creative Commons license, unless indicated otherwise in a credit line to the material. If material is not included in the article's Creative Commons license and your intended use is not permitted by statutory regulation or exceeds the permitted use, you will need to obtain permission directly from the copyright holder.
To view a copy of this license, visit https://creativecommons.org/licenses/by/4.0/

Dr. Matt Tomkins

Department of Geography
Arthur Lewis Building
The University of Manchester
Oxford Road, Manchester, M13 9PL, UK

Email: matthew.tomkins@manchester.ac.uk

6th May 2026

Dear Reviewers,

Nature Communications Manuscript NCOMMS-26-004319

The impact of canals on urban temperatures: a national-scale assessment

Thank you for your constructive comments on our manuscript. We were happy to address all the comments raised – these are detailed below (indented, blue shading). Line numbers are based on the resubmitted manuscript (including tracked changes).

Reviewer #1 (Remarks to the Author):

This is an interesting topic and worthy of study but I feel the paper needs to be expanded to improve the readability and quality.

Due to the layout of the document with the methodology at the end we are presented with a very brief introduction and then some results that only mention a 'model' without any details. Overall the introduction lacks sufficient detail to prepare the reader for the results - we need a short paragraph at the end of the intro to describe the model and what it does, along with a rationale for this type of model versus say a full microclimate simulation.

> We agree with the reviewer that the manuscript would be improved with additional methodological detail and justification e.g., a rationale for the model type, justification for the choice of reference surface, and detail on the calculation of the key fluxes, as noted in the following comments.
>
> Given the typical structure of a *Nature Communications* article, we believe much of this information would be best placed in the 'Materials and Methods' section, which we have updated where relevant, and signposted to this from the introduction (Line 65). In addition, we have modified the introduction to include further justification for the model type, as recommended above (Lines 61 - 85):
>
>> "In this study, we model the thermal effects of canals on the surrounding environment, which are a little-studied (*14*) but widespread feature of post-industrial urban areas. We perform a national-scale analysis of Great Britain and Ireland, where an extensive urban canal network (Fig. 1) reflects the legacy of the Industrial Revolution (*15*). Our model (see Materials and Methods) quantifies the energy balance of urban canals, incorporating radiative, evaporative, and conductive–convective exchanges (*16*), where energy fluxes associated with water are compared to those of a reference surface (asphalt) to estimate the potential change in air temperature (ΔPT). The model is based upon a simplified representation of land use, incorporating the characteristics of the modelled canals (i.e., geometries, depths) and key features of the surrounding environment (i.e., weather, atmospheric shading by nearby buildings), all of which can play a key role in surface energy balance.
>>
>> This parsimonious approach complements computationally intensive micrometeorological simulations (*17*), which might incorporate additional environmental characteristics (e.g., building materials, vegetation) and/or hydrological–atmospheric

processes, modelled at high spatial (c. 1 – 10 m) and temporal resolutions (time-steps of c. 1 – 5 seconds), but which are typically applied to a small number of sites for limited durations (c. 24 – 48 hours). In contrast, our approach enables simulation of a complete annual cycle at a national-scale, which allows us to assess the cooling potential of urban waterways in the context of new temperature extremes (*18*), and the implications of urban form for heat mitigation (*19*)."

The data in fig 3 is I assume over a year - the axes are poorly labelled.

Axis label updated to "year-month" format, which is now used consistently across Figures 2 – 4.

We have no information about the frequency of measurement.

This information has been added to the caption for Figure 3 as follows (Lines 130 – 131):

"Water temperature was measured at 10 minute (A, D, F), 15 minute (E), or hourly frequency (B, C)."

We also have no information about how well the modelled data maps the monitored data over the course of a day. Does the energy balance model accurately predict surface water temperatures over the course of a day?

Thank you for this suggestion. To investigate this, we adapted our code to allow the user to filter the measured and modelled data to a specific date of interest. We agree with the reviewer that this daily information will be of interest to the reader, so have updated the Supplementary Materials to include examples of daily model performance for representative dates (March, June and September) in a new section (S6 – Short-term model performance). We have also included analysis of hourly model performance, extracting absolute residuals for each hour of the day across the year (Figure S13). New figures are available in the Supplementary Materials, with the following accompanying text (Lines S264 – 378):

"S6 – Short-term model performance

Based on comparison of modelled *wT* and measured *wT* for the annual time-series data (Figure 3), overall model performance is very good, with residuals commonly in the range ±1 − 2°C and Spearman's rank correlation coefficients of 0.57 – 0.81 ($p < 0.01$).

To investigate model performance over shorter timescales, and the degree to which modelled *wT* corresponds to measured *wT* over the course of a day, data were filtered to representative dates in March, June and September (Figures S10 – S12). Relevant statistics were calculated, including absolute median residuals, to provide information on the average deviation of modelled *wT* from measured *wT*, and Spearman's rank correlation coefficients, to evaluate the strength and direction of association. These data provide useful insights into daily model performance, which largely reflects the broader trends observed when the annual time-series was used (Figure 3). For example, model performance is generally improved in June and September (Figures S11 – S12), with evidence of under-prediction in March (Fig. S10).

However, these examples provide only a snapshot of model performance, which might be expected to vary daily or hourly as a function of model parameterisation, the inclusion and correct representation of relevant environmental characteristics, and the quality of the input data. To investigate this further, we calculated absolute residuals for each hour of the day across the year (00:00:00 – 23:00:00, $n = 24$), with the results shown in Figure S13. For the Leeds and Liverpool, Nottingham and Beeston and Huddersfield canals, model performance is consistent across the day (Fig. S13, A, B, E), with absolute median residuals of c. 1 – 1.5°C. For the remaining canals there is evidence of some variability in model performance, with higher absolute residuals in the morning for the Shropshire Union and Coventry canals (Fig. S13, C, F), and in the afternoon for the Bridgewater and Taunton Canal (Fig. S13 D). This variability might reflect additional environmental characteristics or energy fluxes that it was not possible to include at the scale of analysis (e.g., shading by vegetation, heat flux due to bed conduction), or the accuracy of the input meteorological or environmental data. Overall, the consistent performance of many of the models (Fig. S13, A, B, E), as well as often

small residuals (e.g., c. < 1°C for Fig. S13 F), indicates that important energy fluxes are being represented correctly in the energy balance model."

We believe the above analysis and accompanying figures have significantly improved the quality of the validation and will allow readers to make informed decisions about the quality of our work. For the main text, we prefer to retain the validation using the entire annual dataset, as depicted in Figure 3, as this provides a wealth of information on model performance (e.g., presence of under- or over-prediction), without compromising clarity. For example, filtering to individual dates increases the number of measured-modelled comparisons to 2,190 (six sites × 365 days).

Details are lacking in the method but I assume the water is treated as fully mixed when in reality a static waterbody like a canal is likely stratified. As such what confidence do we have that the evaporated, convected and radiated heat from the water surface is accurate?

Our model treats the canal as stratified (i.e., not fully mixed), with each canal depth split into 0.2 m layers. This is now highlighted more clearly in the Materials and Methods on Lines 452 – 459, as included below. This model design reflects the stratification that is likely for a static waterbody, as noted by the reviewer, which should give confidence in our calculation of the related energy fluxes. The correspondence between measured and modelled wT also indicates that the important energy fluxes are represented correctly in the energy balance model.

"The energy exchange between the water surface and surrounding air (Q_n) is dependent upon the surface water temperature, which is influenced by the temperature distribution within the water column. To account for this, the water is modelled as a stratified system, with the canal divided into vertical layers, with corresponding conductive–convective exchanges. This design reflects the stratification that is likely in water bodies with minimal water flow, but the underlying assumptions would be invalid for water bodies with higher flow velocities, and where additional energy fluxes would be required (e.g., Q_{fc})."

Of greatest concern are the statements regarding improvements to thermal comfort. The results presented are all in terms of temperature improvement only. There is no consideration of humidity or radiant temperatures which are also significant aspects of thermal comfort.

The reviewer is correct that our models produce predictions of water-reference temperatures, and in turn, air temperature change (Δ*PT*). This is the focus of much of the discussion. However, we recognise that thermal comfort is also important, and this can incorporate a wider range of environmental characteristics, such as humidity and radiant heat.

To reflect this, we calculated a simplified version of wet-bulb globe temperature (WBGT), a thermal comfort index based on air temperature (°C) and humidity (%). WBGT was calculated using the input meteorological data, and with modified air temperature data, including canal effects (+Δ*PT*), to assess the degree to which canals effect the number of hours of moderate, high and extreme heat risk.

Crucially, however, we did not do so uncritically, and we recognise that humidity is an important component of thermal comfort (Lines 199 – 201). Calculating WBGT (or any other thermal comfort index) with modified temperature *alone*, would lead to an unrealistic appraisal of changing heat risk, especially when changes in humidity are possible (e.g., Moyer and Hawkins, 2017).

In turn, we performed a detailed sensitivity analysis, calculating WBGT for a range of humidity values, as described on Lines 201 – 204, and detailed fully in the Supplementary Materials (S4). This indicated that the canal network would reduce the number of hours of heat risk with moderate increases in humidity (c. ≤10%) but would begin to negatively impact thermal comfort beyond this. Given we anticipate only minor increases in humidity due to the presence of small, narrow waterbodies (Lines 230 – 237), it seems likely that canals are improving thermal comfort within the immediate vicinity of the canal network. In summary, while we have not modelled humidity change explicitly, we have incorporated it in our calculations of thermal comfort.

While radiant heat also influences thermal comfort, this can operate at a different scale to our model i.e., individuals in the same general location might experience different levels of thermal comfort due to variable blocking of solar radiation by vegetation, the built environment or cloud cover. To minimise the number of assumptions required to model this, we preferred a simplified

thermal comfort index which captures the broad drivers of thermal comfort at the city-scale (temperature, humidity).

I also require statements regarding thermal comfort and heat stress reduction to be reframed to air temperature improvements

Related to the above discussion, we could remove statements referring to thermal comfort from the manuscript and focus on air temperature change alone. However, we believe that our calculation of WBGT improves the practical significance of the study, as recognised by reviewer two, and that the analysis is of sufficient rigour (e.g., inclusion of sensitivity testing of humidity) to warrant inclusion.

The use of asphalt as the reference material also serves to improve the stated cooling potential. As a minimum I would like to see a greater justification for the use of asphalt over say concrete or bare soil as the reference material, along with details of how this material was incorporated into the model (depth, conductivity, heat capacity and emissivity).

We agree with the reviewer that further justification of the reference material is needed. Selection of the reference surface undoubtedly influences the degree of warming or cooling, as recognised in the literature (Völker et al. 2013) and noted on Lines 342 - 343. To outline our choice more fully, we have updated Lines 342 – 359 as follows:

> "One key model choice is the selection of the reference surface or site, which has a substantial effect on the strength of the measured thermal impact (*13*). Here, we model the effects of canals on surrounding air temperatures in comparison with an equivalent reference area of asphalt. We regard this as a reasonable assumption for an urban environment, where high emissivity (c. 0.7 – 0.9), and low albedo (c. 0.1 – 0.2) materials are common. Moreover, given the narrow, elongate shape of the studied canals, we regard asphalt roads as the most likely alternative land-use. However, we recognise that use of reference materials with differing characteristics (e.g., albedo, emissivity, thermal conductivity, density) would influence the magnitude of ΔPT. For example, we would anticipate reduced ΔPT when the reference site includes greenspace (*14*), as increased shading, evapotranspiration and albedo would influence sensible and latent fluxes and likely suppress thermal contrasts between the water and reference surface. Similarly, use of construction materials with higher albedos (e.g., concrete) would be expected to reduce ΔPT. In turn, reference surface or site characteristics are likely to be a key confounding variable in any meta-analysis (*13*)."

The above text includes discussion of concrete, which is a reasonable alternative, and would likely reduce ΔPT due to the higher albedo. A comparison with bare soil would require incorporation of different processes (e.g., changing albedo and energy fluxes in wet *vs.* dry soil), and is less applicable to an urban environment, where most available land is utilised. Comparison could also have been made to a reference site including greenspace (i.e., grass, trees). Much like bare soil, this would require modelling of additional energy fluxes (e.g., evapotranspiration) and would lead to alternative research questions i.e., do greenspaces or bluespaces have a greater impact on the urban thermal environment? While this is a valid and interesting question, it complicates assessment of the role of bluespaces alone, which is the aim of our analysis, and which is best served by comparison with a 'typical' urban material.

As outlined previously, and in relation to the following comment, we have included much more methodological detail, including further discussion of the reference surface modelling (e.g., Lines 494 – 500). It should also be noted that all parameters related to the reference surface (i.e., depth, conductivity, heat capacity, emissivity) are included in the publicly available code (`params.py`).

The methodology lacks detail - the basic heat balance equation is introduced but there is no detail on how the constituents such as Qe, Qh or Qr are calculated.

As noted above, we have added information throughout the Materials and Method section Including more information on the modelling of Qe, Qh and Qr as follows (Lines 467 – 500):

> **Net radiation**

For modelling Q_r, incoming solar radiation was estimated using PySolar (*55*), incorporating the effects of cloud cover (*56*):

$$I = 1 - 0.00243C - (4.24 \cdot 10^{-5})C^2 \tag{3}$$

where *I* = fraction of clear sky irradiance expected and *C* = percentage of cloud cover in the sky. Non-solar radiative thermal absorption (*a*) was modelled incorporating cloud height (*K*), cloud cover (*C*), air temperature (T_a) and relative humidity (*RH*), following (*57*), using a fixed value of *K* = 0.06 to represent cloud heights > 5 km:

$$a = [1 + KC^2] \cdot 8.78 \cdot 10^{-13} \cdot {T_a}^{5.852} \cdot RH^{0.07195} \tag{4}$$

Thermal emissions (*e*) were estimated following the Stefan–Boltzmann law, where ε = surface emissivity, σ = Stefan–Boltzmann constant and T_s = surface temperature.

$$e = \varepsilon \sigma {T_s}^4$$

Latent flux

For modelling Q_e, the evaporation–condensation rate was calculated using a Penman-type empirical equation following (*54*):

$$E_v = 0.165 \cdot (0.8 + (0.864 \cdot U) \cdot (E_w - E_a) \tag{5}$$

where E_v = evaporation/condensation rate, *U* = wind speed, E_w = saturated vapour pressure at water surface temperature and E_a = vapour pressure at air temperature.

Sensible flux

For modelling Q_h for water, a formulation developed by (*58*) was used:

$$Q_h = p_a c_{pa}(c + d \cdot U)(T_a - T_s) \tag{6}$$

where p_a = air density and c_{pa} = the specific heat of air. The empirical constants *c* and *d* are defined as $c = 1.99 \cdot 10^{-3}$ (m s^{-1}) and $d = 2.13 \cdot 10^{-3}$, following (*58*).

For modelling the equivalent flux Q_h for the reference surface (via forced convection), heat transfer was calculated using an empirical correlation for laminar flow over a flat plate as follows:

$$Q_h = h \cdot (T_a - T_s) \tag{7}$$

where *h* = heat transfer coefficient, calculated as follows:

$$h = 0.664 \cdot T_c \cdot Pr^{\frac{1}{3}} \cdot U^{\frac{1}{2}} \cdot v^{-\frac{1}{2}} \tag{8}$$

where T_c = thermal conductivity, *Pr* = Prandtl number and *v* = kinematic viscosity.

Overall this is a good paper on a worthy topic which will contribute to the field but we require significantly more information within the paper itself so there reader can make informed decisions.

Thank you very much for your appraisal of our work, and the recommendations above, which have improved the methodological clarity.

Reviewer #2 (Remarks to the Author):

The influence of urban blue spaces on microclimate regulation is comparatively underrepresented in the literature compared to the extensive work on urban green infrastructure. This gap is particularly evident in narrow urban waterways such as canals, which have received far less attention than lakes.

Furthermore, modeling approaches are still less common than studies based on remote sensing or field measurements, but they offer various advantages, such as the ability to capture day and night assessments. Against this background, the present study makes a valuable contribution to this field of research. The focus on canal systems in urban areas and the inclusion of WBGT as an indicator of thermal comfort for pedestrians further increase the relevance and practical significance of the study. A notable strength of the study is the validation of the modeled water temperatures using measurements from multiple monitoring sites, which increases confidence in the modeling approach. Another important strength is the simulation of a complete annual cycle, which allows for the assessment of the seasonal variation as well as the nocturnal thermal effects of canals – an aspect that is often neglected, as many remote sensing-based studies primarily capture daytime conditions.

Overall, the methodology is well-founded and described in sufficient detail to ensure reproducibility. The reported results appear plausible and are consistent with the values reported in the literature. The conclusions are generally supported by the modeling results presented.

> Thank you for your summary of our work.

The following points may help improve clarity and interpretation:

The section discussing the impact on thermal comfort based on the WBGT index contains direct modeling results and might be better placed in the "Results" section.

> We agree with the reviewer and have moved the description of the WBGT approach and results to a new section in the Results, entitled "Effects on thermal comfort" (Lines 181 - 204), with the interpretation of the WBGT results retained in the discussion. We have also added some additional content to the latter, for further context (Lines 230 – 240):
>
> > "Using the simplified WBGT thermal comfort index (*26, 27*), incorporating canal effects (+Δ*PT*) was sufficient to reduce the number of hours of moderate, high and extreme heat risk, under scenarios of moderate increases in humidity (c. ≤10%). While bluespaces have been shown to increase humidity (*18*, *34*), we anticipate only small differences within the immediate vicinity of the canal network (c. 20 – 50 m). Larger differences have been recorded elsewhere (c. +15 – 20% above background rates), but only for significantly larger bluespaces e.g., the c. 1.3 km wide Yangtze River, Wuhan (*35*). While further work would be required to model humidity change explicitly, or include more complex thermal comfort indexes, such as Physiological Equivalent Temperature (*36*), the above approach indicates that canals have the potential to reduce heat risk."

The discussion should emphasize that the input data on land use are greatly simplified and that the absence of vegetation probably intensifies thermal contrasts and may lead to an overestimation of cooling values and heat stress (WBGT). This simplification does not invalidate the results but should be more clearly framed as a modelling assumption and limitation.

> We agree with the reviewer, and have added extra detail to the introduction to justify the model approach (Lines 68 – 85):
>
> > "The model is based upon a simplified representation of land use, incorporating the characteristics of the modelled canals (i.e., geometries, depths) and key features of the surrounding environment (i.e., weather, atmospheric shading by nearby buildings), all of which can play a key role in surface energy balance.
> >
> > This parsimonious approach complements computationally intensive micrometeorological simulations (*17*), which might incorporate additional environmental characteristics (e.g., building materials, vegetation) and/or hydrological–atmospheric processes, modelled at high spatial (c. 1 – 10 m) and temporal resolutions (time-steps of c. 1 – 5 seconds), but which are typically applied to a small number of sites for limited durations (c. 24 – 48 hours). In contrast, our approach enables simulation of a complete annual cycle at a national-scale, which allows us to assess the cooling potential of urban waterways in the context of new temperature extremes (*18*), and the implications of urban form for heat mitigation (*19*)."
>
> In our original submission, we had acknowledged the role of vegetation in the discussion as follows:

"While we regard this as a reasonable assumption for an urban environment, we would anticipate reduced ΔPT when the reference site includes greenspace (14)"

To emphasise this further, we have updated this as follows (Lines 349 – 355):

"However, we recognise that use of reference materials with differing characteristics (e.g., albedo, emissivity, thermal conductivity, density) would influence the magnitude of Δ*PT*. For example, we would anticipate reduced Δ*PT* when the reference site includes greenspace (*14*), as increased shading, evapotranspiration and albedo would influence sensible and latent fluxes and likely suppress thermal contrasts between the water and reference surface."

The discussion could further highlight the relevance of this modeling approach for urban planning applications. For practical implementation, future model development should incorporate more detailed representations of land use, including low vegetation and tree cover.

Thank you for this suggestion. We agree that the planning context is important, as this was one of the original drivers of the research i.e., how should bluespaces and adjacent land areas be best managed to improve thermal comfort?

Given the scope of this study, which is focused on bluespace impacts alone, we have touched on this briefly on Lines 359 – 364, but agree that inclusion of additional environmental characteristics warrants further study, with potential benefits for planning:

"Future applications of the model could incorporate a range of reference materials and/or more detailed environmental characteristics to further guide urban planning. For example, modelling of vegetation cover could be used to assess net Δ*PT* e.g., the combined thermal impacts of blue– and greenspaces (50). Similarly, modifying the reference surface to reflect varying land use in the vicinity of each modelled canal might provide further information on spatial variability in Δ*PT*."

Reviewer #3 (Remarks to the Author):

This manuscript conducts a national-scale quantitative assessment of the cooling effects of urban canal sections across Great Britain and Ireland by constructing a physical energy balance model. The research is innovative in its integration of physical modeling with temporal and seasonal analysis, providing a valuable quantitative reference for the microclimate effects of urban canals. However, the rationality of the model's simplification in the transition from water temperature simulation to air temperature effect needs further refinement. The current model architecture may significantly overestimate the cooling benefits of canals, and supplementary validation experiments for key physical processes are required.

Thank you for your appraisal of our work. In addition to addressing the comments outlined below, we have included further justification of the model approach in the Introduction (Lines 68 – 85) as follows:

"The model is based upon a simplified representation of land use, incorporating the characteristics of the modelled canals (i.e., geometries, depths) and key features of the surrounding environment (i.e., weather, atmospheric shading by nearby buildings), all of which can play a key role in surface energy balance.

This parsimonious approach complements computationally intensive micrometeorological simulations (*17*), which might incorporate additional environmental characteristics (e.g., building materials, vegetation) and/or hydrological–atmospheric processes, modelled at high spatial (c. 1 – 10 m) and temporal resolutions (time-steps of c. 1 – 5 seconds), but which are typically applied to a small number of sites for limited durations (c. 24 – 48 hours). In contrast, our approach enables simulation of a complete annual cycle at a national-scale, which allows us to assess the cooling potential of urban waterways in the context of new temperature extremes (*18*), and the implications of urban form for heat mitigation (*19*)."

Regarding the final comment above ("supplementary validation experiments for key physical processes are required"), our model utilises existing physically based equations and empirical

formulae, many of which have already been verified by the original authors. For example, we used PySolar for modelling incoming solar radiation, which has been validated using data from the United States Naval Observatory (USNO). To model the effects of cloud cover on incoming radiation, we used a formulation proposed by Nevins and Apell (2021), which was developed based upon three years of observations from the National Renewable Energy Laboratory (NREL), split into training (~68%), validation (~11%) and testing sets (~21%). For modelling the evaporation–condensation rate, a Penman-type empirical equation was used following Webb and Zhang (1997), where results had been validated using on-site measurements (via an evaporation pan). Within this context, our validation is focused on the overall model results i.e., the correspondence between measured and modelled water temperatures, rather than the individual model components.

(1) The literature review on the cooling effects of urban blue spaces is insufficient, with the cited references being as old as 2021. Several relevant articles on the cooling effects of rivers have been published in recent years, and the statements and references regarding certain controversies should be updated accordingly, especially since the author already addressed a recent survey (Fricke et al., 2024) in the discussion

Thank you for these suggestions, which we have reviewed and cited where relevant. The review of urban blue-green spaces by Wang et al. (2025) was particularly useful, as this complemented new discussion on Lines 359 – 364:

> "Future applications of the model could incorporate a range of reference materials and/or more detailed environmental characteristics to better guide urban planning. For example, modelling of vegetation cover could be used to assess net ΔPT e.g., the combined thermal impacts of blue– and greenspaces (*50*). Similarly, modifying the reference surface to reflect varying land use in the vicinity of each modelled canal might provide further information on spatial variability in ΔPT."

We have also referenced Fricke et al. (2024) more widely in the discussion, touching on some of the "prospects for future research" noted in their conclusions (e.g., Line 314, Line 328).

(2) The core design of the model relies on an energy balance equation, validating the model by comparing measured and simulated water temperatures through a series of idealized simplifications regarding underlying surfaces, meteorology, and urban morphology. Is using a "single asphalt reference surface" an oversimplification of the energy model design? It remains unclear whether the effects would change if the surrounding reference surfaces were grass or trees, or if the 3D building density of the surrounding city affects the cooling distance and intensity. Detailed descriptions of different underlying surface compositions and supplementary experiments are missing.

We agree with the reviewer that further justification of the asphalt reference is necessary here, as also noted by reviewer one. To outline our choice more fully, we have updated Lines 342 – 359 as follows:

> "One key model choice is the selection of the reference surface or site, which has a substantial effect on the strength of the measured thermal impact (*13*). Here, we model the effects of canals on surrounding air temperatures in comparison with an equivalent reference area of asphalt. We regard this as a reasonable assumption for an urban environment, where high emissivity (c. 0.7 – 0.9), and low albedo (c. 0.1 – 0.2) materials are common. Moreover, given the narrow, elongate shape of the studied canals, we regard asphalt roads as the most likely alternative land-use. As a final consideration, selection of a material which amplifies thermal contrasts allows us to assess the potential thermal impacts of canals *in isolation*. However, we recognise that use of reference materials with differing characteristics (e.g., albedo, emissivity, thermal conductivity, density) would influence the magnitude of ΔPT. For example, we would anticipate reduced ΔPT when the reference site includes greenspace (*14*), as increased shading, evapotranspiration and albedo would influence sensible and latent fluxes and likely suppress thermal contrasts between the water and reference surface. Similarly, use of construction materials with higher albedos (e.g., concrete) would be expected to reduce ΔPT. In turn, reference surface or site characteristics are likely to be a key confounding variable in any meta-analysis (*13*)."

We agree with the reviewer that including different types of greenspace (i.e., grass, trees) would influence the magnitude of the thermal impact. However, this would require modelling of additional energy fluxes (e.g., evapotranspiration) and would lead to alternative research questions i.e., do greenspaces or bluespaces have a greater impact on the urban thermal environment? While this is a valid and interesting question, it complicates assessment of the role of bluespaces *alone*, which is the aim of our analysis, and which is best served by comparison with a 'typical' urban material.

With respect to building density, we agree that this is an environmental characteristic that is likely to influence the magnitude of ΔPT, for example due to differential shading (included in our model) or wind-tunnel effects. To investigate the effects of building density fully would require a different research strategy, probably utilising sensitivity testing of different degrees of urbanisation i.e., buildings heights, densities and proximities to the canal network. While this an interesting avenue for further research, we believe it is outside of the scope of this study.

As outlined previously, we have included more methodological detail, including further discussion of the energy balance modelling for the reference surface (e.g., Lines 494 – 500). It should also be noted that all parameters related to the reference surface (i.e., depth, conductivity, heat capacity, emissivity) are included in the publicly available code (`params.py`).

(3) The abstract of this paper claims to have developed a novel energy balance model. Compared to existing research on energy equation models over the years, what are the specific breakthroughs and contributions of this study? Furthermore, are there any significant differences in the energy balance equations constructed when targeting canals versus lakes for calculating cooling effects?

We thank the reviewer for this critique, which has allowed us to reflect on the framing of the methodology.

The energy balance model design and implementation is "new" in that it has been built from scratch (in Python) and has been tailored to the specifics of the analysis (i.e., investigating bluespace impacts at scale). Using a stricter definition for "novelty", however, the model is built upon existing physically based equations and verified empirical formulae from published literature, which are now described more fully in the Materials and Methods. While these individual components are not "new", we regard their combination as novel, capturing the key processes operating at our scale of analysis.

What is certainly "novel" is the scale and application of our model, which has enabled a national-scale perspective on canal thermal impacts and the simulation of an entire annual cycle, which we have now emphasised in the introduction (i.e., Lines 76 – 85) and in the conclusions (Lines 406 – 408).

(4) In the results, the interpretation of the canal's morphological parameters primarily uses "area" as the main variable. The narrow and elongated state of canals, along with other morphological parameters, are not elaborated upon in detail. The study offers limited insights into how water body types, different scales, and various shapes of canals contribute to the explanatory power of the cooling effect.

Our model incorporates three morphological parameters for each canal section: *area*, *width*, and *depth*. Our model treats the canal as stratified (i.e., not fully mixed), where each canal *depth* is split into 0.2 m layers. The *area* of each section influences the magnitude of the modelled energy fluxes. The *width* of each section determines the modelled cooling distance, as noted in the methods (Lines 543 – 546) and expanded upon more fully in the Supplementary Materials (S2). In turn, while the interpretation in the results does focus on area (e.g., comparison of the energy balance of water with a reference *area* of asphalt), other morphological parameters are included.

The reviewer is correct that we have not analysed different water body morphologies. While we agree that this is an interesting avenue for further study, we do not believe it is within the scope of this paper. While there will inevitably be some variability in the size and shape of the studied water bodies, most canals are of a similar morphology, with relatively little variability in width ($p5$ = 9.2, $p95$ = 27.2 m) and depth (c. 1 – 2 m), for example, reflecting their common Industrial heritage.

(5) In the discussion, the author clearly explains the simplification of the energy equation and the humidity-rising effect caused by evaporation, which is commendable. However, many processes and mechanistic effects have not been fully accounted for. Calculating △PT and the cooling-effect-corrected WBGT through extreme simplification is problematic, as these are precisely the elements that need accurate measurement in current research and findings.

In this paper we chose to produce a parsimonious model to predict water-reference energy balance. The correspondence between modelled and measured *wT* for the validation sites (Figure 3), indicates that the model has captured the important energy fluxes and environmental characteristics. However, it is certainly true that many processes and mechanistic effects have not been accounted for, particularly when changes in water-reference energy balance are used for predicting air temperature change. As a result, we have been careful to frame our results cautiously and analyse them critically where possible.

For example, in the introduction we framed the modelled changes in temperature as *potential* changes in air temperature (Δ*PT*) (Line 68). Similarly, the first section of the discussion is entitled "Cooling *potential* of urban canals" (Line 210) to highlight this framing to the reader. We acknowledged the importance of selecting the reference surface or site (Lines 342 – 359), which can amplify or suppress the measured thermal impact. In the Materials and Methods, we also discussed the potential energy flows within the urban boundary and canopy layers, and other factors which might influence Δ*PT* (Lines 510 – 516).

Where possible, we have also included sensitivity testing to improve the rigour of our analysis. For example, this has included testing of the effects of the bluespace cooling distance on modelled Δ*PT* (Supplementary Materials S2), as well as the impacts of humidity on WBGT (Supplementary Materials S4).

While the model could be refined in future to incorporate additional processes, the corresponding increase in computational complexity must be balanced against our ability to investigate the canal network at a national-scale and for an entire annual cycle. In our view these are the key strengths of our analysis. As noted by the reviewer, we hope our work provides a "valuable quantitative reference for the microclimate effects of urban canals". This might include comparison of our work with computationally intensive micrometeorological simulations, or field-based measurements (e.g., Hathway and Sharples, 2012), which will either verify or challenge our predictions. This framing is important to communicate to the reader, so we have updated the manuscript on Lines 398 – 405 as follows:

> "While the choice of cooling distance, and other key model attributes described above, will inevitably influence model performance, the modelled ΔPT values appear reasonable within the context of published literature (45), showcase behaviour that is consistent with on-site measurements and physical principles e.g., daytime cooling, nighttime warming (10), and provide a valuable quantitative reference for the microclimate effects of urban canals, which can be tested further using micrometeorological simulations or field-based measurements."

(6) Supplementary Material S1 presents a "full energy equation" but omits latent heat flux. Because the study spans full seasonal cycles, including winter conditions where water surfaces may freeze, further adjustments to the energy equation parameters are necessary to account for these scenarios.

In Supplementary Material S1, subplot A illustrates the correspondence between modelled water temperatures and measured water temperatures when the full energy balance equation is used ($Q_n = \pm Q_r \pm Q_e \pm Q_h$ i.e., radiative ± latent ± sensible fluxes). This approach underpins all the results discussed in the main text. Subplot B illustrates the correspondence when latent fluxes ($\pm Q_e$) are removed. This figure was included to demonstrate the important role of latent fluxes, even if latent flux is not used directly for calculating air temperature change, as argued in the main text (Lines 533 – 539).

We agree with the reviewer that freezing and other modifications to energy balance could be incorporated into the model, as we noted in the main text (Lines 117 – 122). While this may be important in certain scenarios, this would require a significant increase in model complexity for, in our view, a relatively minor improvement in model performance. While there are some

differences between measured and modelled water temperatures during the winter period, for example some under-prediction during the later months of 2022, deviations in winter do not appear systematically larger than during other periods of the year (Figure 3). While incorporating these additional fluxes could improve model fit in some cases, it would also require additional assumptions (e.g., depth of freezing layer) and inclusion of complex feedbacks (e.g., changes to convection), which if parameterised incorrectly, might degrade rather than improve the performance of the model. Given the scale and duration of our analysis, we prefer to produce a parsimonious model, which focuses on the energy fluxes that can be modelled with the greatest confidence and which return the best performance across the annual cycle.

(7) The empirical fit in Panel A of Supplementary Figure S4 looks suspicious. The distribution is highly skewed and appears to rely on only the first and last data points, rather than a proper regression across the full dataset.

Figure S4 A shows regression equations between bluespace widths and corresponding cooling distances. The grey regression line (y = 1.4136x + 31.3869) includes data from Hathway and Sharples (2012), Murakawa et al. (1991) and Park et al. (2019). To the best of our knowledge, these are the only studies which have directly measured the relationship between the *width* of a bluespace and the cooling distance. By comparison, there are many studies which have assessed the relationship between bluespace *areas* and cooling distances (Figure S4 B). However, these measurements are not appropriate for the studied canals, as argued in the supplementary text (Lines S-66 – S72).

While conducting a regression across the full dataset is possible (grey line), we argue that this produces unrealistic values for narrow waterbodies due to the intercept term (Lines S-86 – 89) i.e., even a canal of 0 m width would still have a cooling distance of >30 m. Instead, we prefer to predict the cooling distance using data from Hathway and Sharples (2012) and Murakawa et al. (1991) alone, which produces more realistic results for the narrow canals of Great Britain and Ireland (y = 1.5126x + 6.7227).

We agree with the reviewer that additional measurements of bluespace geometries and cooling distances would improve the robustness of the regression and should be the object of further study. While alternative regressions and changes to the predicted cooling distances would influence the magnitude of the modelled warming or cooling, this would not fundamentally change our interpretations e.g., seasonal or short-term effects, given the results of our sensitivity analysis (Fig. S5).

(8) Regarding Fig. S7 (Scatter plot of region attributes vs. median daytime △PT), why is the nighttime situation not supplemented?

Given the relative magnitude of the daytime and nighttime Δ*PT* effects, our focus was on the former. However, we agree with the reviewer that investigation of nighttime effects is warranted. We have included an additional figure in the supplementary materials to address this (Fig. S8) and the following text to describe the results (Lines S-146 – 151):

> "Regional differences in median nighttime Δ*PT* are comparatively small, ranging from c. +0.1 – 0.3°C, and are correlated with average humidity, as shown in Figure S8. Increased nighttime Δ*PT* (i.e., enhanced warming) under scenarios of higher humidity is likely explained by reduced evaporative cooling and a supressed latent heat flux, resulting in higher water temperatures, and a greater temperature difference with respect to the reference surface."

(9) Could Section S5 be expanded to include city-level breakdowns of population size and the share of residents living within 1 km of canals? This would make it clearer how many people actually benefit from the cooling effect and how these benefits are distributed across the study area.

Thank you for the suggestion. We have added a new table (Table S2) to the supplementary materials, summarising the population within 1 km by region (15-minute walking distance) and as a percentage of the total urban area, focusing on the largest regions for clarity (population ≥ 75,000). The following text is included to describe the approach and results (Lines S-217 – 234):

"To investigate how accessibility varied across the study area, the canal network was subset by region and the above methodology repeated. Region-specific population totals are reported in Table S2, focusing on those with an accessible population >75,000. For each region, we calculated the total population within corresponding local authority areas to estimate the share of residents with access to the canal network (~15-minute walking distance). It should be noted that these estimates are sensitive to the calculation of the urban area population, which will vary as local authorities are included or excluded, or if more a spatially precise urban classification is used. Based on this approach, canals in Birmingham and London are accessible to the largest populations (c. 1.1 million individuals), and together account for c. 37% of the total accessible population. When considered in the context of local authority populations, canals in Birmingham are serving the largest share (c. 45%), reflecting the size and distribution of the canal network, which stretches across much of the urban area. In contrast, canals are only easily accessible to a comparatively small share of the London population (c. 13%) due to the absence of canals from large parts of the city. Other regions, such as Leeds, Stoke-on-Trent, and Burnley and Nelson, also serve a large share of the local authority population (≥ 40%), reflecting the co-occurrence of canals and residential areas within each region."

Reviewer #3 (Remarks on code availability):

I have carefully reviewed the code and found no obvious errors.

Thank you for your review of our code.

Additional changes made by the authors

[1] Tomkins and Huck have included their university research group affiliation in the author details (MCGIS, *Mapping, Computing and Geographical Information Science)*

[2] We have also acknowledged former students at The University of Manchester (Sophie Taylor, Joe Chambers, Barry Johnston, Niall Coogan), for their contributions to the wider project.

[3] We removed a small number of words from the abstract to meet the 200-word maximum (Lines 15 – 32)
